# A complex suite of loci and elements in eukaryotic type II topoisomerases determine selective sensitivity to distinct poisoning agents

**DOI:** 10.1093/nar/gkz579

**Published:** 2019-07-09

**Authors:** Tim R Blower, Afif Bandak, Amy S Y Lee, Caroline A Austin, John L Nitiss, James M Berger

**Affiliations:** 1 Johns Hopkins University School of Medicine, Department of Biophysics and Biophysical Chemistry, Baltimore, MD 21205, USA; 2 Department of Molecular & Cell Biology, University of California, Berkeley, Berkeley, CA 94720, USA; 3 Institute for Cell and Molecular Biosciences, Faculty of Medical Sciences, Newcastle University, Newcastle upon Tyne, NE2 4HH, UK; 4 Biopharmaceutical Sciences Department, University of Illinois College of Pharmacy, 1601 Parkview Avenue, N310, Rockford, IL 61107, USA

## Abstract

Type II topoisomerases catalyze essential DNA transactions and are proven drug targets. Drug discrimination by prokaryotic and eukaryotic topoisomerases is vital to therapeutic utility, but is poorly understood. We developed a next-generation sequencing (NGS) approach to identify drug-resistance mutations in eukaryotic topoisomerases. We show that alterations conferring resistance to poisons of human and yeast topoisomerase II derive from a rich mutational ‘landscape’ of amino acid substitutions broadly distributed throughout the entire enzyme. Both general and discriminatory drug-resistant behaviors are found to arise from different point mutations found at the same amino acid position and to occur far outside known drug-binding sites. Studies of selected resistant enzymes confirm the NGS data and further show that the anti-cancer quinolone vosaroxin acts solely as an intercalating poison, and that the antibacterial ciprofloxacin can poison yeast topoisomerase II. The innate drug-sensitivity of the DNA binding and cleavage region of human and yeast topoisomerases (particularly hTOP2β) is additionally revealed to be significantly regulated by the enzymes’ adenosine triphosphatase regions. Collectively, these studies highlight the utility of using NGS-based methods to rapidly map drug resistance landscapes and reveal that the nucleotide turnover elements of type II topoisomerases impact drug specificity.

## INTRODUCTION

**Figure 1.**
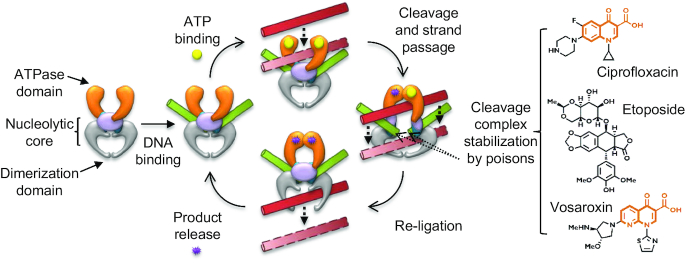
Type II topoisomerases undergo an ATP-dependent cycle of DNA-binding, strand capture, cleavage, strand passage and re-ligation to pass a double-stranded DNA (in red) through another double-stranded DNA (in green). ATP (yellow circles) is converted to ADP (purple stars) in the process. Topoisomerase poisons such as ciprofloxacin, etoposide and vosaroxin bind at the nucleolytic core's two opposing DNA cleavage sites to block strand passage and re-ligation. Drug structures under investigation here are shown, with the similar quinolone cores of ciprofloxacin and vosaroxin highlighted in orange.

Type II DNA topoisomerases are ubiquitous cellular enzymes that generate transient breaks in chromosomes to pass one double-stranded DNA segment through another (1). The strand-passage activity of type II topoisomerases is required for multiple essential processes, including transcription, DNA replication and chromosome segregation (2,3). The type IIA topoisomerase subclass catalyzes strand passage by sequentially opening and closing three dissociable subunit-subunit interfaces—termed ‘gates’—in an adenosine triphosphate (ATP)-controlled manner (Figure 1) (4–7). A challenge faced by type II topoisomerases during strand passage is a need to repetitively cleave and re-ligate DNA while avoiding the accidental formation of persistent genotoxic double-strand breaks (8).

Budding yeast encode one type IIA topoisomerase, yTOP2, whereas mammals, including humans, possess two isoforms, hTOP2α and hTOP2β (9). The two human isoforms appear to have partially independent functions (3,10). Human TOP2α is expressed in proliferating cells (11,12), where it participates in DNA replication, chromosome condensation and sister chromatid separation (10,13,14). By contrast, hTOP2β is expressed throughout all cell types, including terminally differentiated cells (15). In contrast to the roles of hTOP2α, hTOP2β has been found to act as an activator of transcription, cleaving DNA at transcriptional start sites (16–18); the activity of hTOP2β has also been proposed to lead to neuronal stimulation and memory formation (19). The impact of DNA cleavage by human type IIA topoisomerases must be tightly controlled, as TOP2-induced chromosomal breakages can lead to neurological diseases and therapy-related cancers (20,21).

Type IIA topoisomerases are clinically-validated targets for a number of antibacterial and anticancer agents (22). Although the overall architectures of the prokaryotic and eukaryotic type IIA topoisomerases are similar (6,23–30), small-molecule inhibitors exist that can selectively target one group over another. For example, fluoroquinolones such as ciprofloxacin are potent antibacterials that comprise nearly a quarter of the $10 billion US antibiotic market (31). Fluoroquinolones act as ‘poisons,’ intercalating between DNA bases at the site of topoisomerase-dependent cleavage (26–28,32) (Figure 1), inducing persistent double-strand breaks that lead to DNA fragmentation and cell death (33). Although generally considered quite selective, some newer-generation fluoroquinolones, such as gatifloxacin and moxifloxacin (which are used in the treatment of tuberculosis) have been reported as displaying host toxicity (34,35). In contrast, vosaroxin (formerly SNS-595 or voreloxin), is a non-fluorinated quinolone derivative that has no reported antibacterial activity, but instead targets eukaryotic topoisomerases (36) and has potential in cancer therapies. For example, phase III trials have shown significantly improved response rates in patients with acute myeloid leukemia (AML) when vosaroxin was combined with cytarabine, compared to placebo and cytarabine combined controls (37,38). Vosaroxin has also been shown to have activity against models of glioblastoma and cervical cancer (39,40).

Despite being the target of multiple drug families in clinical use, current knowledge of how different compounds discriminate between distinct topoisomerase orthologs remains limited. To gain a greater understanding of the factors governing drug selectivity, we undertook a series of mutagenesis screens, using a next-generation sequencing (NGS) approach to identify a more comprehensive picture of topoisomerase variants capable of distinguishing between different quinolone scaffolds. The diversity of the resistance mutant population isolated from this effort was surprisingly rich, and showed that a number of mutations which map outside of the DNA cleavage center (where type II topoisomerase poisons are known to bind) can confer either highly specific or broad-spectrum resistance, even to drugs that were not included in the original selection. Biochemical examination of several enzyme variants confirms the outcomes of our mutagenesis screens and further led us to discover that the susceptibly of human topoisomerases to poisoning can be modulated by the ATPase elements of the enzymes. Interestingly, ciprofloxacin is found to have activity against yTOP2 *in vitro* and kills *Saccharomyces cerevisiae*, suggesting that fluoroquinolones may have unexplored utility as anti-fungal agents.

## MATERIALS AND METHODS

### Yeast *top2^ts^* complementation

Complementation studies were performed in yeast strain *S. cerevisiae* JN394_t2–4_ (41). Plasmids pMJ1 (42) and YEphTOP2β-KLM (43) provided wild-type (WT) hTOP2α and hTOP2β, respectively. The vector-only control (pTRB302) was constructed by digesting pMJ1 with HindIII and re-ligating to exclude *htop2α*. The yTOP2 complementation plasmid (pTRB318) was constructed by replacing the *htop2α* gene in pMJ1 with a *ytop2* ORF encoding the full 1–1428 amino acids, using the MluI-SalI restriction enzyme sites. Competent JN394_t2–4_ cells were prepared as instructed (Sigma-Aldrich, YEAST-1 transformation kit), except the strain was grown at 25°C because growth of strains carrying the *top2–4* allele is greatly reduced at 30°C in liquid culture. Transformants of JN394_t2–4_ were selected on solid SC-URA plates, incubated at 30°C. The choice of media used for complementation (SC-URA versus YPD) was found to impact the growth-inhibiting capacity of topoisomerase II poisons, with ciprofloxacin and vosaroxin becoming significantly less toxic against hTOP2α and yTOP2 compared to etoposide when grown on SC-URA (Supplementary Figure S1A). For complementation studies, triplicate overnight cultures were therefore grown in YPD media at 30°C, 200 rpm and adjusted to a final OD_600_ of ∼1. The 10-fold serial dilutions were made and spotted onto YPDA plates containing either no drugs, 500 μM ciprofloxacin (Sigma-Aldrich) in ddH_2_O, 100 μM etoposide (Sigma-Aldrich) in dimethyl sulfoxide (DMSO) or 100 μM vosaroxin (Sunesis Pharmaceuticals Inc.) in DMSO. Once the spots had dried, plates were incubated at room temperature as the permissive temperature and 34°C for non-permissive temperature. Viable counts and images were taken after three days of growth.

Additional mutants of pMJ1 and pTRB318 were generated by site-directed mutagenesis, using either the QuikChange (Agilent) or ‘round-the-horn’ (44) technique. All new constructs were sequenced in full prior to use in complementation studies.

### Chemical mutagenesis and mutant plasmid selection

Plasmids pMJ1 and pTRB318 were subjected to hydroxylamine mutagenesis (45). Hydroxylamine solution was prepared by dissolving 0.35 g of hydroxylamine hydrochloride and 0.09 g NaOH in 5 ml of ice-cold ddH_2_O. Ten microgram of plasmid DNA was mixed with 500 μl of hydroxylamine solution and incubated at 42°C for 18.5 h. The reaction was then cleaned using a polymerase chain reaction (PCR) clean-up kit (Macherey-Nagel) and the sample was eluted in 50 μl of ddH_2_O. Approximately 800 ng of mutagenized DNA was used to transform freshly prepared competent JN394_t2–4_. Transformed cells were initially divided in two pools and plated on both solid SC-URA plates incubated at room temperature and YPDA plates incubated at 34°C. The colony count at the non-permissive temperature was reduced ∼30–35%, implying mutagenesis had been successful and that the remaining 65–70% of colonies contained mutant plasmids still able to complement the *top2–4* growth defect at 34°C. Vosaroxin-resistant mutants of hTOP2α and ciprofloxacin-resistant mutants of yTOP2 were selected by plating transformants on YPDA supplemented with 100 μM vosaroxin or 500 μM ciprofloxacin, respectively, and incubating at 34°C for 3 days. Ciprofloxacin-resistant mutants of yTOP2 were then picked back onto fresh YPDA plates containing 500 μM ciprofloxacin and onto a second plate containing 100 μM vosaroxin (together with WT yTOP2 negative control patches), then again incubated at 34°C for 3 days. The patches were compared after two days to identify colonies that were ciprofloxacin-resistant, vosaroxin-sensitive and ciprofloxacin-resistant, vosaroxin-resistant.

For the small-scale screens, plasmids were extracted from selected colonies using glass beads and brief boiling, as described (46). The topoisomerase II ORFs were then amplified from the isolated plasmids by PCR and these amplicons were sequenced directly, to avoid a further round of potential mutation during passage through an *Escherichia coli* host. To prevent the influence from any secondary mutations on the isolated plasmid or within the original mutant colony, identified mutations were then re-constructed by site-directed mutagenesis and tested for drug sensitivity as described above. Three of seventeen mutants had a different resistance profile to that expected from patching (Supplementary Figure S1B and C). This suggested a low error rate in this initial screening and patching process. For the larger screen, multiple patching steps were performed in order to reduce this error rate. It was initially unclear how an opal nonsense mutation that was isolated (W653STOP), could confer drug resistance. As both frame-shifted and truncated variants of this construct could not complement (Supplementary Figure S1D), we speculate that there is a low level of enzyme produced from W653STOP sufficient to promote cell growth, but that also results in fewer DNA damage events.

For the large-scale screens, colonies were patched to fit ∼100 colonies per plate. For the hTOP2α vosaroxin-resistant screen, 380 colonies were patched from transformant plates onto YPDA containing 100 μM vosaroxin. For the yTOP2 ciprofloxacin-resistant, vosaroxin-sensitive and ciprofloxacin-resistant, vosaroxin-resistant screens, once colonies with the correct phenotype had been identified, they were patched in groups of approximately 100 colonies back onto YPDA plates. There were 537 ciprofloxacin-resistant, vosaroxin-sensitive and 210 ciprofloxacin-resistant, vosaroxin-resistant isolated in this manner. All plates were again incubated at 34°C for 3 days. To extract the mutant plasmids, all colonies on a single plate were scraped together and the surface was washed with a further 3 ml of sterile ddH_2_O and cells were re-suspended by vortexing. A 1.5 ml aliquot of this re-suspension was pelleted by centrifugation. DNA was then extracted from these cells as described above. The final DNA was eluted in 120 μl of ddH_2_O, at a concentration of between 1 and 3.5 μg.μl^−1^. The mutant ORFs were amplified from each of these DNA pools by PCR. Gel extracted products of the expected size were taken forward for NGS.

### Library preparation and computational analysis

Gel-extracted DNA products were fragmented to 200–500 nt size distribution using NEBNext dsDNA Fragmentase (NEB). Following end repair and A-tailing, Illumina-compatible adapters were ligated, and libraries were amplified by PCR. Following library cleanup using AmpPure beads, multiplexed libraries were sequenced in one lane of a HiSeq2000 (Illumina) sequencing system in normal run mode. Low quality raw Illumina reads were trimmed or removed using the FASTX Toolkit (http://hannonlab.cshl.edu/fastx_toolkit/), and read-through into Illumina adapters was removed with Cutadapt (http://code.google.com/p/cutadapt/). Reads were mapped to provide ORF sequences of *htop2α* and *ytop2* using Bowtie (47), allowing only unique alignments and a maximum of 1 mismatch per read. A python script was developed to perform additional analysis. First, only reads that contained mismatches were extracted, corresponding to ∼10% of the ∼50 million reads per sample. Next, extracted reads were sorted for confidence. The ‘quality’ scores from the Illumina process were used to decide which mismatches were real rather than simply error during sequencing—only scores of 38 and above were kept, which corresponded to 99.98% confidence in base calling. At last, the remaining mismatches were extracted and converted to amino acid mutations together with a calculation of the number of supporting reads. Mutations were ranked according to the number of supporting reads and the cut-off was set as the top 100 mutations. This corresponded to a cut-off of 4213 reads for the vosaroxin-resistant mutants of hTOP2α and 2471 reads and 3870 reads for the ciprofloxacin-resistant, vosaroxin-sensitive and ciprofloxacin-resistant, vosaroxin-resistant mutants of yTOP2, respectively. Since the sequence library was from a pooled PCR product, the numbers of reads obtained do not correlate with colony numbers, but merely reveal those mutations that are most highly represented in the pool and that are therefore unlikely to be artifacts.

To map the mutations, sequence alignments were first produced with Clustal Omega (48) and where needed, mapped using Consurf (49). Structural figures were generated using PyMol (50).

### Topoisomerase II cloning, expression and purification

PCR-amplified full-length topoisomerase genes were inserted by LIC, ligation-independent cloning (51), into 12UraB (Addgene #48304), a modified version of pRS426 (52). The resulting plasmids (hTOP2α-12UraB, hTOP2β-12UraB and pTRB378 for yTOP2–12UraB) contained galactose-inducible fusions with an N-terminal tobacco etch virus (TEV) protease-cleavable hexahistidine tag. Residues 409–1177 of yTOP2 were amplified and similarly cloned into 12uraB to produce the yeast core enzyme expression construct. Residues 431–1193 of hTOP2α and residues 447–1206 of hTOP2β were amplified and cloned by LIC into the pET-based vector plasmid 2BT (Addgene #29666), generating IPTG-inducible fusions with an N-terminal TEV protease-cleavable hexahistidine tag, which could be expressed in *E. coli*. Mutant full-length constructs were generated by PCR amplification of the mutant ORFs from complementation plasmids and LIC cloning into 12UraB, followed by re-sequencing. Core enzyme mutants were generated by site-directed mutagenesis of the WT core constructs, as described above.

Overexpression of the full-length WT and mutant constructs, and the yeast core enzyme, was performed in *S. cerevisiae* strain BCY123, grown in complete supplement mixture dropout medium lacking uracil (CSM-URA), supplemented with 2% (vol.vol^−1^) lactic acid and 1.5% (vol.vol^−1^) glycerol as carbon sources. Cells were grown at 30°C, 150 rpm, to an OD_600_ of 0.8–1.0 and induced by the addition of 20 g.l^−1^ galactose. After a further 6 h growth at 30°C, 150 rpm, cells were harvested by centrifugation (4500 × *g*, 15 min, 4°C) then re-suspended in yeast buffer (250 mM NaCl, 1 mM EDTA), and frozen drop-wise in liquid nitrogen.

Frozen cells were cryogenically lysed using a Spex 6870 freezer mill, with 15 cycles of 1 min grinding followed by 1 min of cooling. The resulting powder was thawed in A300 [20 mM Tris–HCl pH 8.5, 300 mM KCl, 20 mM imidazole pH 8.0, 10% (vol.vol^−1^) glycerol with protease inhibitors (1 μg.ml^−1^ pepstatin A, 1 μg.ml^−1^ leupeptin and 1 mM PMSF)] and clarified by centrifugation (17 000 × *g*, 20 min, 4°C). The lysate supernatant was passed over an A300-equilibrated HisTrap HP column (GE Healthcare) then washed with 30 ml of A300 and 25 ml of A100 [20 mM Tris–HCl pH 8.5, 100 mM KCl, 20 mM imidazole pH 8.0, 10% (vol.vol^−1^) glycerol with protease inhibitors], and connected to an Akta Explorer FPLC (GE Healthcare). A HiTrap S HP column (GE Healthcare) was connected in series and equilibrated with a further 5 ml of A100. The columns were then washed with 25 ml B100 [20 mM Tris–HCl pH 8.5, 100 mM KCl, 200 mM imidazole pH 8.0, 10% (vol.vol^−1^) glycerol with protease inhibitors] to elute the tagged protein onto the S column and a further 15 ml of A100 to reduce imidazole levels. A gradient was then applied, reaching 100% buffer C [20 mM Tris–HCl pH 8.5, 500 mM KCl, 10% (vol.vol^−1^) glycerol with protease inhibitors] over 25 min. Peak fractions were assessed by sodium dodecyl sulphate-polyacrylamide gelelectrophoresis (SDS-PAGE), collected and concentrated in Amicon 100-kDa-cutoff concentrators (Millipore). TEV protease was added to the concentrated samples and incubated at 4°C overnight. This mixture was then passed over a second HisTrap HP column equilibrated and washed with buffer D [20 mM Tris–HCl pH 8.5, 500 mM KCl, 20 mM imidazole pH 8.0, 10% (vol.vol^−1^) glycerol] to remove uncleaved product and protease. The flowthrough was collected and concentrated, then separated by gel filtration using an S400 column (GE Healthcare) equilibrated in sizing buffer [20 mM Tris–HCl pH 7.9, 500 mM KCl, 10% (vol.vol^−1^) glycerol]. Peak fractions were pooled and concentrated. Final samples were combined with a one-third volume of storage buffer [20 mM Tris–HCl pH 7.9, 500 mM KCl, 70% (vol.vol^−1^) glycerol], quantified by NanoDrop (ThermoScientific) and aliquots were snap frozen to be stored at −80°C prior to use.

The hTOP2α and hTOP2β WT and mutant core enzymes were overexpressed in *E. coli* strain Rosetta 2 pLysS (EMD Millipore) by growing cells in 2x YT, at 37°C and 150 rpm until an OD_600_ ∼0.3. At this point, the temperature was reduced to 16°C and cells were left to grow to an OD_600_ of 0.6–1.0. They were then induced with 0.5 mM IPTG and left to grow overnight (20 h) at 16°C. Cells were harvested by centrifugation (4500 × *g*, 20 min, 4°C), then re-suspended in buffer A800 [20 mM Tris–HCl pH 7.9, 800 mM NaCl, 30 mM imidazole pH 8.0, 10% (vol.vol^−1^) glycerol with protease inhibitors] and frozen drop-wise in liquid N_2_. When needed, cells were thawed on ice and lysed by four cycles of sonication (15 s burst with 2 min rest on ice). Lysates were clarified by centrifugation (17 000 × *g*, 30 min, 4°C) and the supernatant was passed over a HisTrap HP column (GE Healthcare), equilibrated in A800. Samples were washed in five column volumes of A800 and a further 10 column volumes of A400 [20 mM Tris–HCl pH 7.9, 400 mM NaCl, 30 mM imidazole pH 8.0, 10% (vol.vol^−1^) glycerol with protease inhibitors]. Protein was then eluted with B400 [20 mM Tris–HCl pH 7.9, 400 mM NaCl, 500 mM imidazole pH 8.0, 10% (vol.vol^−1^) glycerol with protease inhibitors]. The eluted samples were concentrated in Amicon 30-kDa-cutoff concentrators (Millipore). TEV was added and the sample was dialysed against 1 L of A400 overnight at 4°C. This mixture was then passed over a second HisTrap HP column equilibrated and washed with buffer A400. The flowthrough was collected and concentrated, then separated by gel filtration using an S300 column (GE Healthcare) on a Bio-Rad DuoFlow system, equilibrated in sizing buffer [50 mM Tris–HCl pH 7.9, 500 mM KCl, 10% (vol.vol^−1^) glycerol]. Peak fractions were pooled and concentrated. Final samples were combined with a one-third volume of storage buffer [20 mM Tris–HCl pH 7.9, 500 mM KCl, 70% (vol.vol^−1^) glycerol], quantified by NanoDrop (ThermoScientific) and aliquots were snap frozen to be stored at −80°C prior to use.

### Preparation of plasmid DNA substrates

Negatively supercoiled pSG483 (2927 bp), a pBlueScript SK+ (Agilent) derivative containing an Nb.BbvCI site, was prepared from *E. coli* XL-1 blue cells (Agilent) using a maxiprep kit (Macherey-Nagel). A portion of this sample was treated with BamHI (NEB) to form linear plasmid. Another portion of this sample was nicked with Nb.BbvCI (NEB) and an aliquot was removed to make a nicked pSG483 stock. A further aliquot was ligated with T4 DNA ligase (NEB), to form relaxed pSG483. All plasmid forms were purified by phenol/chloroform extraction and ethanol precipitation prior to use.

### Plasmid assays

Aliquots of full-length test protein were thawed on ice for 10 min. These were then serially diluted in 2-fold steps using protein dilution buffer [50 mM Tris pH 7.5, 500 mM KOAc, 2 mM MgOAc, 1 mM dithiothreitol (DTT), 50 μg.ml^−1^ bovine serum albumin (BSA) and 10% (vol.vol^−1^) glycerol], down to 156.25 nM of topoisomerase II dimers. A reaction master mixture was made containing four parts diluted enzyme, five parts 4× reaction buffer [40 mM Tris pH 7.5, 38.4 mM MgOAc, 4 mM DTT, 100 μg.ml^−1^ BSA and 32% (vol.vol^−1^) glycerol] and one part of a 500 ng.μl^−1^ solution of substrate (either negatively supercoiled or relaxed) pSG483 plasmid DNA. The master mixture was incubated on ice for 5 min. Drug titrations were prepared by mixing 2 μl of an appropriate drug dilution (or 2 μl of solvent for ‘zero drug’ controls’) with 1 μl of 20 mM ATP and 7 μl of ddH_2_O. These 10-μl drug mixtures were then added to 10-μl aliquots of the full reaction mixture on ice, quickly transferred to 37°C and incubated for 30 min. Final reaction conditions were 60 nM full-length dimers, 15 nM supercoiled pSG483, variable drug content (or solvent), 1 mM ATP, 20 mM Tris pH 7.5, 100 mM KOAc, 10 mM MgOAc, 1.2 mM DTT, 35 μg.ml^−1^ BSA and 10% (vol.vol^−1^) glycerol. Following incubation the reactions were first quenched with 2 μl of stopping buffer [5% (wt.vol^−1^) SDS and 125 mM EDTA], followed by adding 1 μl of 12 mg.ml^−1^ proteinase K and a further incubation at 37°C for 30 min. Samples were stored on ice until immediately before gel loading, whereupon a 6× agarose gel loading dye was added and the samples were warmed to 37°C for 5 min. Samples were separated by electrophoresis in 1.4% (wt.vol^−1^) TAE agarose gels (50 mM Tris–HCl, pH 7.9, 40 mM NaOAc and 1 mM EDTA pH 8.0 running buffer), with and without ethidium bromide (0.5 μg.ml^−1^) for 6–15 h at 2–2.5 V/cm. To visualize the DNA, gels run without ethidium bromide were poststained with 0.5 μg.ml^−1^ ethidium bromide in TAE buffer for 30 min, destained in TAE buffer for a further 30 min and exposed to UV illumination. Supercoiled relaxation/cleavage assays were performed with the core enzymes as per the supercoil relaxation assays above, except the final concentration of core dimers used in the assays was 50 with 10 nM of supercoiled pSG483. All reactions were ran in triplicate and gel images were analyzed using ImageJ (53), and data were plotted using Prism (GraphPad Software).

### Ligation assay

Reactions were constructed as for the above supercoiling assays, except there was no protein present, no ATP and the DNA substrate used was 300 ng of nicked pSG483. Following 30 min incubation at 37°C, 1 μl of 20 mM ATP was added together with 1 μl of T4 DNA ligase (NEB). These samples were then incubated for a further 30 min at 37°C. Reactions were stopped, separated by gel electrophoresis and visualized as above.

## RESULTS

### Drug-resistant type II topoisomerase mutations can be broadly acting or highly selective for specific poisons

**Figure 2.**
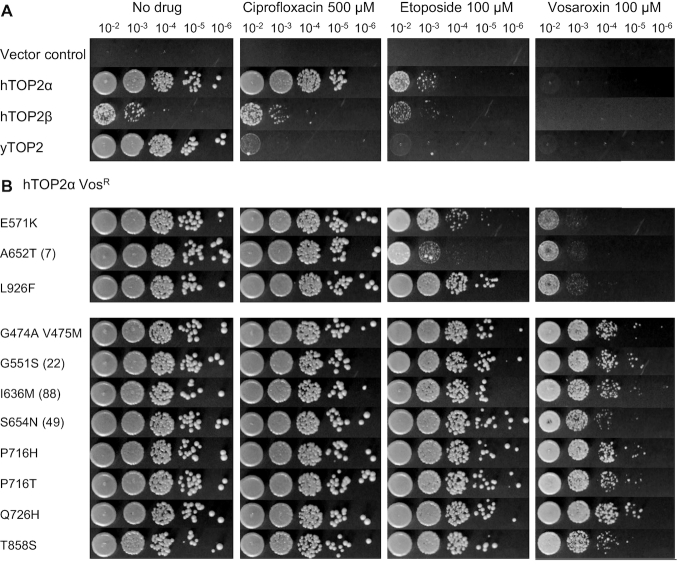
Screening for poison-resistant type II topoisomerases. (**A**) Viable counts of JN394_t2–4_ transformed with complementation constructs, serially diluted as indicated and plated at non-permissive temperature (34°C), in the absence and presence of topoisomerase poisons. Images are representative of triplicate cultures. (**B**) Viable counts of JN394_t2–4_ complementation by hTOP2α mutants. Numbers in parentheses indicate the rank position of the mutation if it falls within the top 100 hits from the later NGS screen.

We first set out to investigate how eukaryotic type II topoisomerases discriminate between different poisons by performing a genetic screen for drug-resistant variants. Assays that selected for resistant variants were carried out using the *S. cerevisiae* strain JN394_t2–4_ (41), which contains a temperature-sensitive allele for yTOP2 and is deficient in the repair of DNA double strand breaks due to a deletion of the *RAD52* gene. At a non-permissive temperature for the *top2–4* allele of 34°C, both hTOP2α and yTOP2 fully complemented growth, as assessed by viable counts (42), whereas hTOP2β provided only an intermediate level of complementation (Figure 2A). These complementation behaviors were consistent with prior studies (43). The sensitivity of the complemented yeast was then tested against three topoisomerase poisons: two quinolone derivatives, vosaroxin and ciprofloxacin, and the anticancer epipodophyllotoxin, etoposide (Figure 1). Ciprofloxacin had no impact on the growth of JN394_t2–4_ when the cells contained hTOP2α and hTOP2β expressing plasmids, but the drug strongly inhibited the growth of cells expressing yTOP2 (Figure 2A). Vosaroxin and etoposide disrupted cell growth regardless of the complementing topoisomerase (Figure 2A).

Given the comparatively weak complementation behavior of hTOP2β, we elected to mutagenize only the hTOP2α and yTOP2 complementing plasmids for use in resistance screens. Following hydroxylamine treatment of plasmid DNA (54) and transformation of JN394_t2–4_, functional vosaroxin-resistant (Vos^R^) alleles of hTOP2α were selected by growth at the non-permissive temperature on YPD plates containing 100 μM vosaroxin. Plasmid DNA was extracted from a subset of Vos^R^ colonies and the *hTOP2α* alleles were sequenced to identify potential mutations. Each identified mutation was re-introduced into the WT complementing plasmid by site-directed mutagenesis, followed by re-transformation and verification of drug sensitivity phenotypes (Figure 2B). All mutant plasmids tested appeared Vos^R^ to varying degrees. Three different single point mutations in hTOP2α (E571K, A652T and L926F) each caused a 100-fold increase in viable cell count when grown with 100 μM vosaroxin in comparison to WT hTOP2α (Figure 2A and B); the hTOP2α^E571K^ mutation has been identified previously in a screen for m-AMSA resistance (55). A second set of single and double hTOP2α mutations (G474A/V475M, G551S, I636M, S654N, P716H, P716T, Q726H and T858S) provided greater resistance to vosaroxin, increasing viable cell counts 10 000-fold (Figure 2B). All mutations tested also conferred resistance to etoposide (Etop^R^), despite an absence of selection against the drug, with hTOP2α^E571K^ and hTOP2α^A652T^ again conferring lower resistance (interestingly, hTOP2α^L926F^ proved highly Etop^R^, but only provided low-level resistance to vosaroxin) (Figure 2B). While performing the screen with hTOP2α, attempts were made to identify mutations that might provide susceptibility to ciprofloxacin. A total of 2500 colonies were screened by negative selection, but no ciprofloxacin-sensitive isolates were detected.

**Figure 3.**
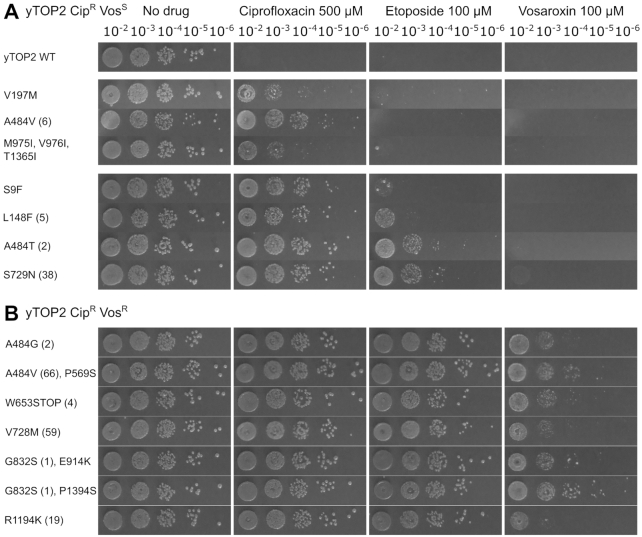
Mutant yTOP2 alleles can discriminate between topoisomerase poisons. (**A**) Viable counts of JN394_t2–4_ complemented with yTOP2 Cip^R^Vos^S^ mutants. (**B**) Viable counts of JN394_t2–4_ complemented with yTOP2 Cip^R^Vos^R^ mutants. Numbers in parentheses indicate the rank position of the mutation if it falls within the top 100 hits from the later NGS screen.

Since yTOP2 proved susceptible to ciprofloxacin (Figure 2A), this topoisomerase was further used to screen for mutant alleles that might discriminate between different quinolone poisons; i.e. changes that conferred resistance to ciprofloxacin, vosaroxin or both. Approximately 28 000 transformants complemented for growth at non-permissive temperature by a pool of mutant yTOP2 expression plasmids were plated out on 500 μM ciprofloxacin to select for functional mutant alleles that conferred drug-resistance. Transferring these colonies onto fresh plates containing 500 μM ciprofloxacin confirmed 363 colonies (∼1.30%) as Cip^R^. When tested for vosaroxin resistance by transferring colonies onto plates containing 100 μM vosaroxin, 45% had a ciprofloxacin- and vosaroxin-resistant (Cip^R^Vos^R^) phenotype, whereas the remaining 55% were ciprofloxacin-resistant but vosaroxin-sensitive (Cip^R^Vos^S^). In a screen selecting for initial vosaroxin resistance, no colonies were recovered that could be identified as Cip^S^Vos^R^. When assessed for etoposide resistance, the Cip^R^Vos^S^ mutations fell into two classes (Figure 3A). The first, which contained two different single point mutants (yTOP2^V197M^ and yTOP2^A484V^) and a triple mutant (yTOP2^M975I/V976I/T1365I^), conferred ciprofloxacin resistance but had no impact on etoposide sensitivity (Figure 3A). The second contained only single yTOP2 mutations (S9F, L148F, A484T and S729N), and manifest a 100- to 10 000- fold increase in etoposide resistance. All examined mutations that generated vosaroxin resistance also conferred ciprofloxacin and etoposide resistance (Figure 3B). Interestingly, some of the observed mutations were quite selective in their resistance behavior. For example, the yTOP2^A484V^ change produced a Cip^R^ phenotype, whereas a threonine change at this position was Cip^R^Etop^R^ and a glycine change was triply Cip^R^Vos^R^Etop^R^ (Figure 3B). Our screen therefore led to the selection, identification and validation of both broadly resistant and drug-selective yTOP2 mutant alleles.

### Resistance mutations to topoisomerase poisons do not localize to any one enzyme region

Having obtained proof-of-principle for our selection strategy (Figures 2B and 3), we developed an NGS-based approach to screen larger mutant pools (Supplementary Figure S2). Through NGS it was possible to obtain sequence information more rapidly and for a far greater number of colonies than would have been practically feasible using Sanger methods applied to single colonies from classic genetic-screens. Colony collections comprising 374 Vos^R^ hTOP2α mutants, 545 Cip^R^Vos^S^ yTOP2 mutants and 209 Cip^R^Vos^R^ yTOP2 mutants were first isolated by manual picking. Mutant plasmids were then prepared in pools, used to generate PCR-amplified libraries of topoisomerase genes and sequenced by NGS. Mutations were ranked according to the number of reads containing the nucleotide change. The top 100 mutations were selected as a cut-off and were taken from totals of 13 781 Vos^R^ hTOP2α mutations, 12 873 Cip^R^Vos^S^ yTOP2 mutations and 12 871 Cip^R^Vos^R^ yTOP2 mutations. Each set of 100 mutants comprised 46, 36 and 46% of the total number of reads for each phenotypic class, respectively. This conservative cut-off ensured that we examined only those hits which occurred relatively frequently (>2400 reads) in comparison to a long tail of mutants with low numbers of reads (Supplementary Figure S3).

**Figure 4.**
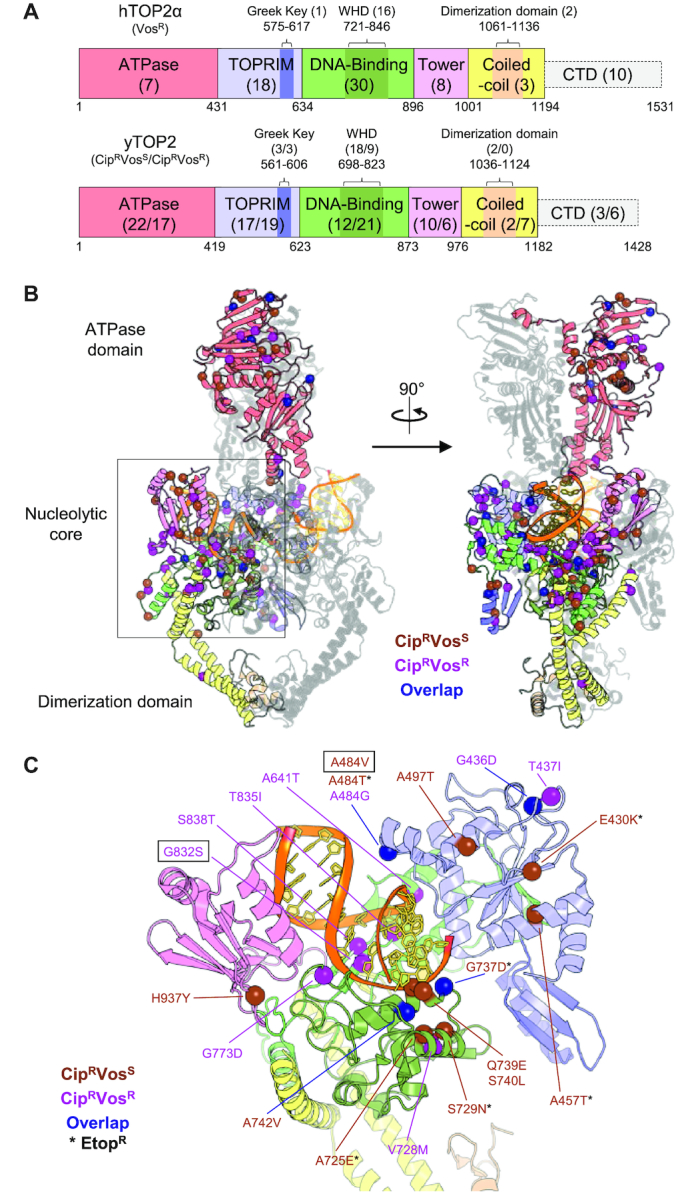
The top 100 hits from the NGS screens for poison-resistant topoisomerase II alleles are dispersed throughout the topoisomerase structure. (**A**) Linear schematic of hTOP2α (upper) and yTOP2 (lower). Each domain is labeled and described by bordering residue numbers. In the case of the Greek key, WHD and dimerization domains, these are embedded within larger regions. Numbers in parentheses indicate the number of mutations from the top 100 hits found within each domain, not including those found in an embedded domain (where applicable). With yTOP2, the first number in parentheses refers to the hits from the Cip^R^Vos^S^ screen and the second number relates to the Cip^R^Vos^R^ screen. (**B**) Overview of yTOP2, with one monomer colored as per (A) and the second monomer shown in transparent gray. DNA backbone and bases are shown in orange and wheat, respectively. Residues identified within the top 100 hits of the Cip^R^Vos^S^ screen are indicated by brown alpha-carbon spheres, residues identified within the top 100 hits of the Cip^R^Vos^R^ screen are shown as purple alpha-carbon spheres and residues that overlap both screens are shown as blue alpha-carbon spheres. A 90° rotation of yTOP2 is also shown. (**C**) Tilted view of the boxed region in (B), with the ATPase domain and the partner protomer/DNA fragments removed for ease of visualization. Residues identified within the top 100 hits of the yTOP2 screens and tested in Figures 3 and 5 are shown and colored as in (B). Mutations that were additionally Etop^R^ are marked with an asterisk. Boxed mutations are those used for later *in vitro* testing. (B and C) were adapted from PDB entry: 4GFH.

**Figure 5.**
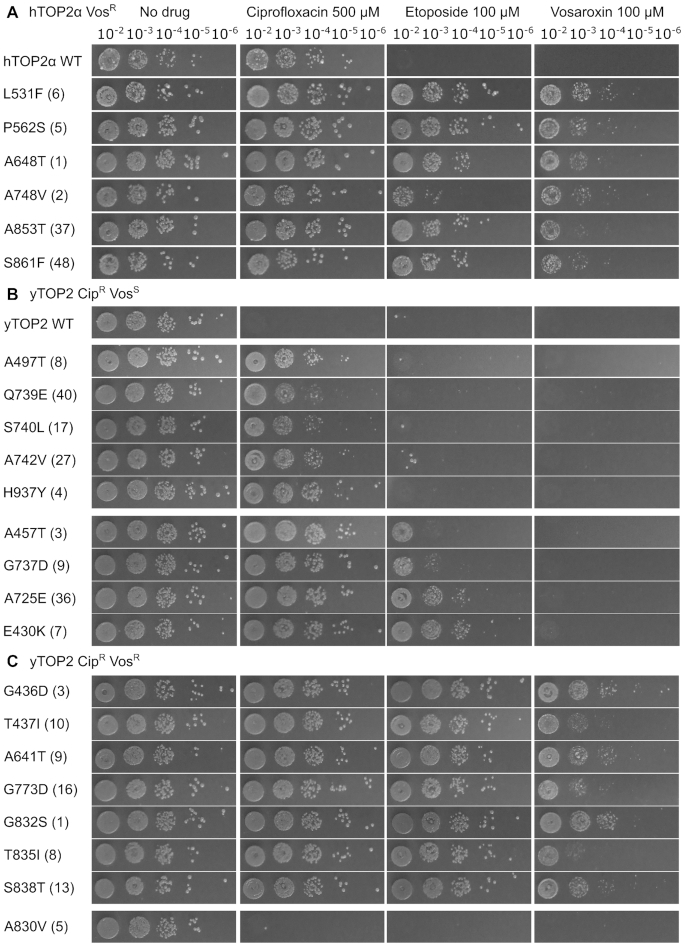
NGS screen-identified mutations were confirmed by phenotypic testing. (**A**) Viable counts of JN394_t2–4_ complemented with hTOP2α Vos^R^ mutants. (**B**) Viable counts of JN394_t2–4_ complemented with yTOP2 Cip^R^Vos^S^ mutants. (**C**) Viable counts of JN394_t2–4_ complemented with yTOP2 Cip^R^Vos^R^ mutants. Numbers in parentheses indicate the rank position of the mutation within the top 100 hits of the NGS screen.

Surprisingly, the top 100 mutations found from each NGS screen turned out to be broadly distributed throughout the topoisomerase coding sequence (Figure 4A; Supplementary Figure S4 and Table S1). For hTOP2α, 46 of the observed mutations mapped to the DNA-binding domain and winged-helix domain (WHD), 19 to the TOPRIM and Greek-key domains and a small number to the ATPase and tower domains (7 and 8, respectively). An additional five hits were found in the coiled-coil and primary dimerization domain, and 10 more in the unstructured C-terminal domain (CTD). The distribution for the yTOP2 mutants was similar, except that a slightly smaller number of substitutions fell within the DNA-binding domain and WHD (40 for Cip^R^Vos^S^ and 30 for Cip^R^Vos^R^) and a greater number mapped to the ATPase region (22 for Cip^R^Vos^S^ and 17 for Cip^R^Vos^R^) (Figure 4A). The structures of yTOP2 (29) and hTOP2α (23,24) were used to map the overall distribution of the top 100 hits (Figure 4B and Supplementary Figure S5). Taking into account read-frequency, functional regions, position of known drug-binding residues and sequence conservation (Figure 4C and Supplementary Figure S6), 23 mutants were chosen in order to confirm the phenotypes of hits from our NGS screen. The selected mutations were introduced by site-directed mutagenesis into the parental hTOP2α and yTOP2 complementation plasmids, which were then used to transform JN394_t2–4_ for testing against the three topoisomerase poisons (Figure 5). All mutations identified from the hTOP2α Vos^R^ screen were doubly Vos^R^Etop^R^ (with hTOP2α^A748V^ proving to be only weakly Etop^R^) (Figure 5A), even when the changes fell far outside the known binding site for etoposide (such as hTOP2α^L531F^). From the yTOP2 Cip^R^Vos^S^ screen, mutations fell in two groups, with the single A457T, G737D, A725E or E430K changes each conferring Cip^R^Etop^R^, and the others showing only Cip^R^ (Figure 5B). The mutations within these two groups also appeared to cluster independently of their ranking within the NGS screen (Figure 5B). For the Cip^R^Vos^R^ screen, nearly all mutations tested led to resistance against all three poisons; the one exception was yTOP2^A830V^, which despite ranking fifth in total number of reads for the screen, remained sensitive to all three drugs (this complete lack of resistance suggests that A830V may work in conjunction with one or more secondary mutations within yTOP2) (Figure 5C). Nevertheless, 22 of the 23 tested mutations recapitulated the phenotype expected from the NGS screen, validating the practical utility of this approach for the high-throughput identification of drug-resistance mutations.

### Assessing the biochemical effects of vosaroxin, ciprofloxacin and etoposide on wild-type yTOP2 and hTOP2α versus select resistance mutants

**Figure 6.**
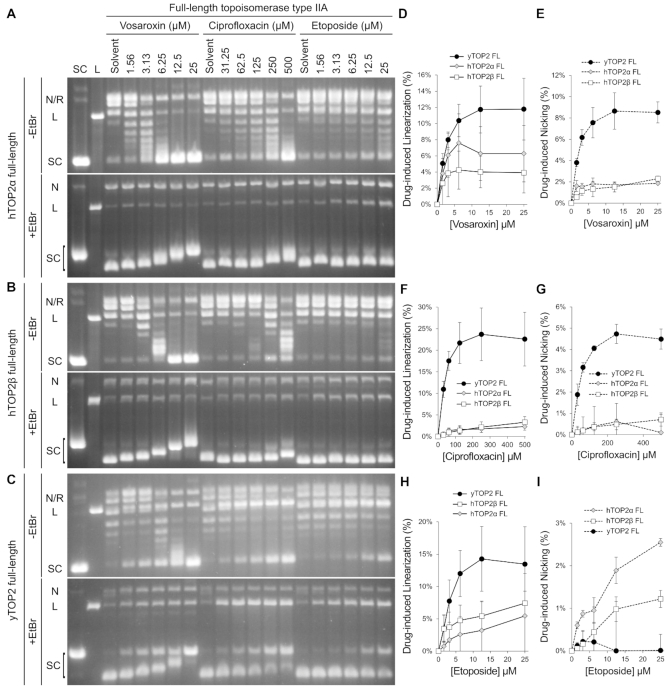
Effects of antibacterial and anticancer topoisomerase poisons on eukaryotic topoisomerases. Relaxation of negatively supercoiled plasmid DNA by WT full-length (**A**) hTOP2α, (**B**) hTOP2β and (**C**) yTOP2, titrated against vosaroxin, ciprofloxacin and etoposide. The positions of linear (L), nicked (N), relaxed (R) and supercoiled (SC) plasmids DNAs are indicated. Images are representative of triplicate comparative reactions, using agarose gels with and without 0.5 μg.ml^−1^ ethidium bromide (EtBr). (**D**–**I**) DNA linearization and nicking by WT full-length (FL) enzymes as induced by (D and E) vosaroxin, (F and G) ciprofloxacin and (H and I) etoposide, quantified by densitometry. Error bars represent the standard deviation of triplicate data.

Before assessing whether the drug-resistant mutants identified by our genetic screens also impart resistance *in vitro*, we first examined the response of purified full-length WT hTOP2α and yTOP2 enzymes to different poisons, alongside full-length hTOP2β as an additional comparison. WT enzymes were tested for their ability to relax negatively supercoiled plasmid DNA in the presence of different drug concentrations, using native agarose gel electrophoresis with and without ethidium bromide to aid the visualization of nicked and linear products (Figure 6). With all three enzymes, vosaroxin induced a low level of DNA nicking and double stranded cleavage and also initially appeared to inhibit supercoil relaxation as well (Figure 6A–E). In agreement with a recent report (56), ciprofloxacin did not appear to stimulate DNA cleavage or nicking by hTOP2α and hTOP2β (Figure 6A, B, F and G), but at the highest concentration tested (500 μM), did begin to inhibit DNA supercoil relaxation (Figure 6A and B). These findings indicate that whereas JN394_t2–4_ cells can still grow in the presence of ciprofloxacin when complemented by hTOP2α and hTOP2β (Figure 2A), sustained elevated levels of ciprofloxacin can nonetheless interfere with DNA supercoil relaxation by these enzymes. In contrast, ciprofloxacin had no discernible impact on supercoil relaxation but readily promoted DNA cleavage and nicking by yTOP2 (Figure 6C, F and G), consistent both with its ability to kill yeast cells in the complementation screen (Figure 2A) and prior biochemical studies of the purified protein (57,58). Etoposide induced an (expected) increase in the amount of linear products produced by all enzymes, albeit more weakly for hTOP2α and hTOP2β as compared to yTOP2 (Figure 6A–C and H). Etoposide also induced low levels of nicking in hTOP2α and hTOP2β, but very little, if any, nicking with yTOP2 (Figure 6C and I).

**Figure 7.**
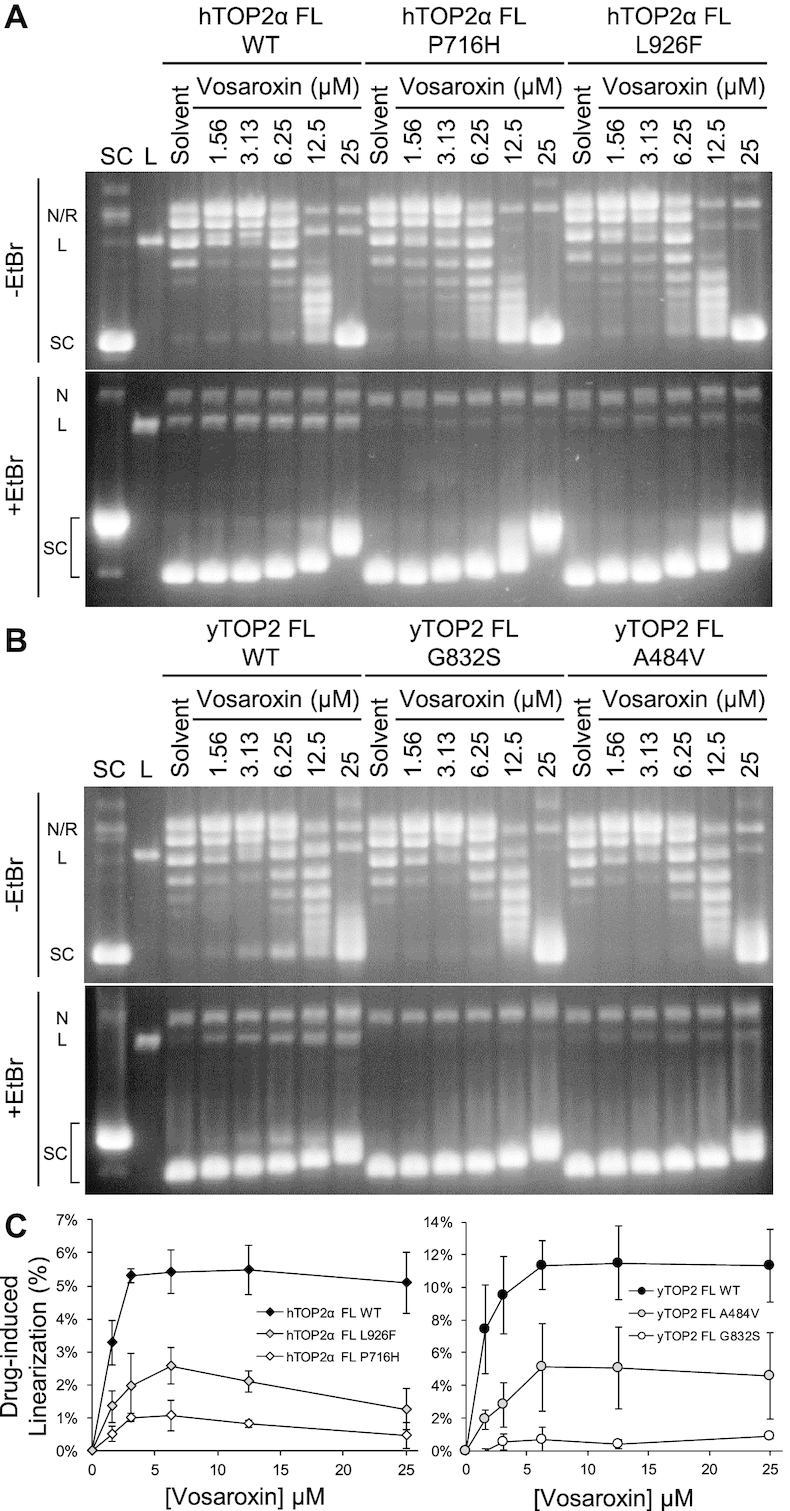
Protection from vosaroxin-induced double-strand breaks by resistance mutations. (**A**) Comparison of WT full-length (FL) hTOP2α supercoiling activity on relaxed plasmid DNA with that of Vos^R^ mutants P716H and L926F, titrated against vosaroxin. (**B**) Comparison of WT full-length (FL) yTOP2 supercoiling activity on relaxed plasmid DNA with that of Cip^R^Vos^R^ mutant G832S and Cip^R^Vos^S^ mutant A484V, titrated against vosaroxin. (**C**) Resistance mutations reduce the levels of drug-induced linearization, as quantified by densitometry. Error bars represent the standard deviation of triplicate data.

Although vosaroxin induced a low level of DNA cleavage with all three enzymes tested (Figure 6A–D), this agent has also been proposed to act as a general inhibitor of topoisomerase II-dependent DNA supercoil relaxation (36). Vosaroxin readily intercalates into DNA (36), an activity that can be seen upon incubating nicked DNA with the drug and sealing the nick with ligase (Supplementary Figure S7A). By itself, vosaroxin has no apparent effect on supercoiled or relaxed plasmid DNA in the absence of topoisomerase (Supplementary Figure S7B). Based on these properties, we hypothesized that the apparent ‘inhibition’ of topoisomerase II-dependent supercoil relaxation seen with vosaroxin (Figure 6A–C) might be misleading, and actually reflects the outcome of active strand passage events by topoisomerase II that trap changes in DNA twist arising from the intercalation of vosaroxin into DNA. To test this idea, we pre-incubated WT and mutant hTOP2α or yTOP2 enzymes with *pre-relaxed* plasmid DNA in the presence of varying amounts of the drug (Figure 7A and B, left third of the gels). In solvent-only control lanes, the pre-relaxed substrate is unaffected by the enzymes. However, as vosaroxin concentrations are increased, the plasmid becomes supercoiled. Comparative reactions run on agarose gels in the presence and absence of ethidium bromide at higher vosaroxin concentrations lead to the production of a plasmid species that co-migrates with the supercoiled control plasmid. These data argue against the possibility that vosaroxin acts as a catalytic inhibitor of supercoil relaxation by type II topoisomerases (36), and instead strongly support the conclusion that vosaroxin's toxicity derives from its action as a topoisomerase II poison and/or as a DNA intercalating agent.

Having established a baseline for drug action on topoisomerase II function *in vitro*, we next examined a pair of drug-resistance mutants for hTOP2α and yTOP2. The hTOP2α^P716H^ and hTOP2α^L926F^ mutants were both Vos^R^ in our genetic screens, with hTOP2α^P716H^ being strongly-resistant and hTOP2α^L926F^ being weakly resistant (Figure 2B). By comparison, for the yTOP2 mutants, the G832S mutation is Cip^R^Vos^R^ (Figure 5C), whereas the A484V mutation is Cip^R^Vos^S^ (Figure 3A). The vosaroxin-free supercoil relaxation activity of these selected topoisomerase II mutants was tested and compared to the respective WT enzyme (Supplementary Figure S8). The hTOP2α^P716H^ construct had no clear difference in activity, whereas hTOP2α^L926F^ had reduced supercoil relaxation activity compared to hTOP2α WT (Supplementary Figure S8A). yTOP2^A484V^ was comparable to yTOP2 WT, while yTOP2^G832S^ had slightly faster activity (Supplementary Figure S8B). The impact of vosaroxin on DNA cleavage by WT and these mutant topoisomerase constructs was then compared in the presence of pre-relaxed plasmid DNA (Figure 7). In accord with the screening data, vosaroxin-induced DNA cleavage was reduced to a greater extent for hTOP2α^P716H^ than for hTOP2α^L926F^, and both were more resistant to the drug than native hTOP2α (Figure 7A and C). Compared to yTOP2 WT, vosaroxin-induced DNA cleavage was also reduced for the Cip^R^Vos^R^ yeast topoisomerase II mutant, yTOP2^G832S^, but less so for the Cip^R^Vos^S^ mutant yTOP2^A484V^ (Figure 7B and C).

**Figure 8.**
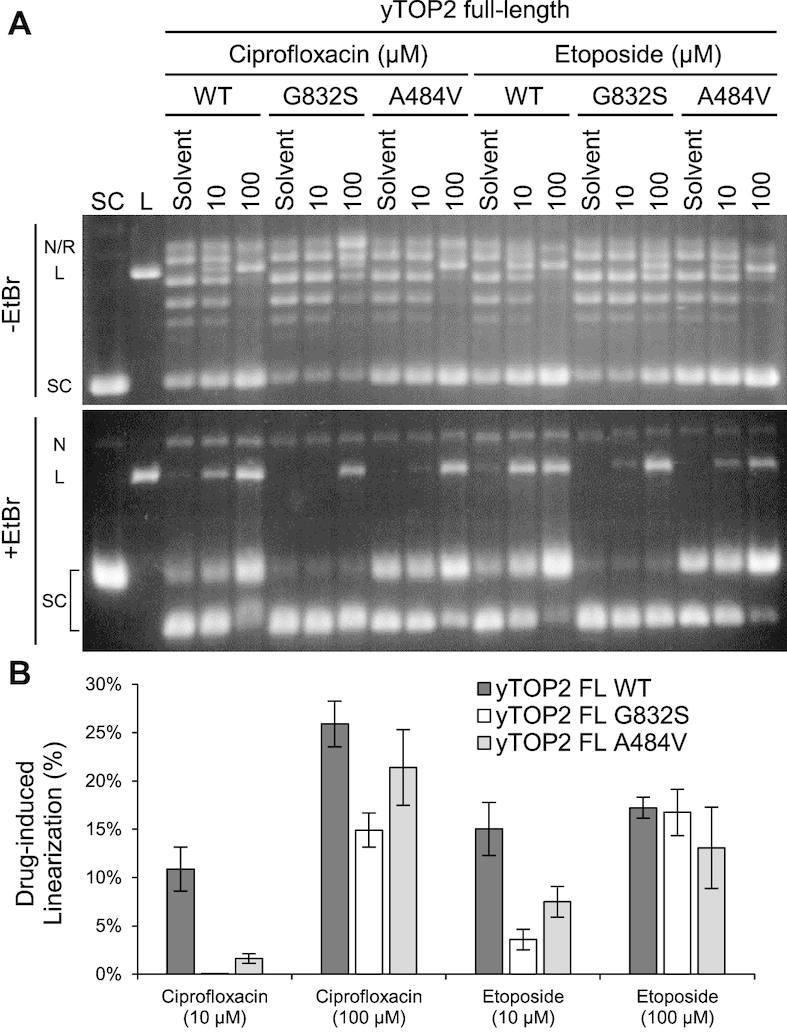
Protection from ciprofloxacin- and etoposide-induced double-strand breaks by resistance mutations. (**A**) Comparison of full-length (FL) WT and mutant yTOP2 plasmid supercoil relaxation activity on negatively supercoiled plasmid substrate, titrated against ciprofloxacin and etoposide. The positions of linear (L), nicked (N), relaxed (R) and supercoiled (SC) plasmids DNAs are indicated. Images are representative of triplicate comparative reactions, using agarose gels with and without 0.5 μg.ml^−1^ ethidium bromide (EtBr). (**B**) Resistance mutations reduce the levels of drug-induced linearization, as quantified by densitometry. Error bars represent the standard deviation of triplicate data.

Next, the yTOP2 mutants were tested for their sensitivity to ciprofloxacin and etoposide during supercoil relaxation (Figure 8). As expected, yTOP2^G832S^ (which is triply resistant to all three drugs tested) formed fewer linear products than WT yTOP2 with either 10 or 100 μM ciprofloxacin, or with 10 μM etoposide (Figure 8). Nevertheless, at the higher (100 μM) etoposide dose, drug-induced linearization was similar between yTOP2 WT and yTOP2^G832S^ (Figure 8). Curiously, the yTOP2^A484V^ mutant (which displayed a Cip^R^Vos^S^Etop^S^ phenotype on plates) appeared modestly resistant to both ciprofloxacin and etoposide at lower concentration (Figure 8). Collectively, these experiments biochemically confirm that NGS screening approaches are capable of identifying new drug-resistant mutant hTOP2α and yTOP2 alleles. These findings also show that resistance to these agents largely manifests as a reduction in cleavage complex formation and that mutations outside the known binding locus for type II topoisomerase poisons can have a significant impact on drug sensitivity without significantly compromising enzyme activity *in vitro*.

### The nucleolytic cores of human type II topoisomerases are susceptible to poisoning by etoposide

**Figure 9.**
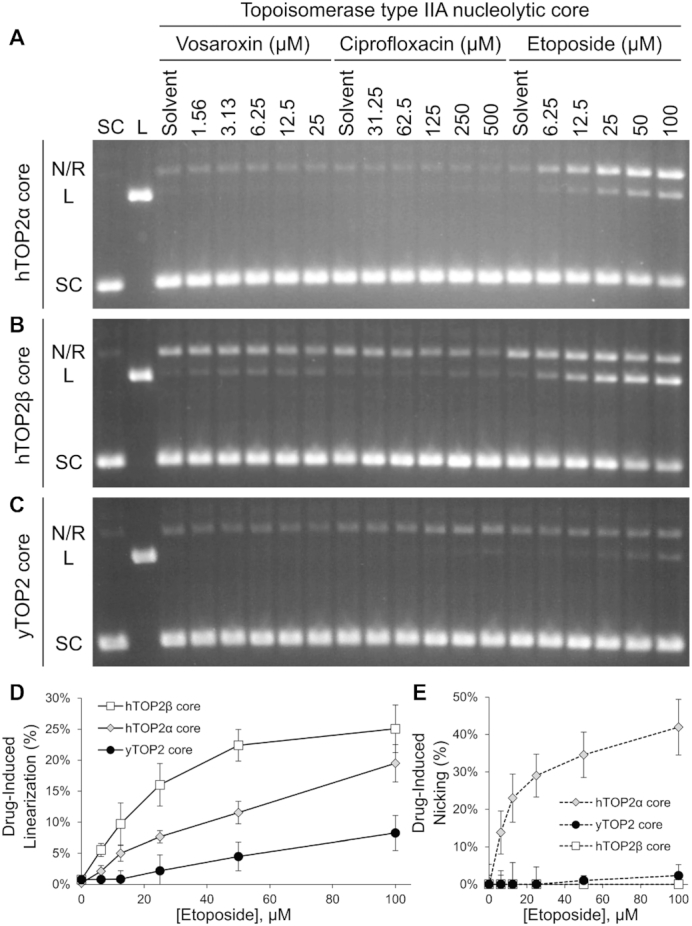
Topoisomerase ATPase domains modulate the impact of topoisomerase poisons. Activity of core (**A**) hTOP2α, (**B**) hTOP2β and (**C**) yTOP2 enzymes on negatively supercoiled plasmid DNA as titrated against vosaroxin, ciprofloxacin and etoposide. The positions of linear (L), nicked (N), relaxed (R) and supercoiled (SC) plasmids DNAs are indicated. Images are representative of triplicate comparative reactions, using agarose gels without ethidium bromide (EtBr). Etoposide-induced linearization (**D**) and nicking (**E**) by core enzymes, as quantified by densitometry. Error bars represent the standard deviation of triplicate data.

Type IIA topoisomerase poisons bind deep within the enzymes’ conserved nucleolytic core, near the dyad of the dimer (Figure 1) (25–28,59). Because the drug-resistance mutations we isolated frequently arose outside of the principle drug-binding locus, we were curious as to how the nucleolytic region alone might respond to poisons. To address this question, the DNA binding and cleavage cores of hTOP2α, hTOP2β and yTOP2 (all of which lack the associated ATPase and unstructured C-terminal domains) were purified and assayed against vosaroxin, ciprofloxacin and etoposide (Figure 9). Etoposide induced plasmid linearization by all three core enzymes in a clear, dose-dependent manner. When testing the full-length enzymes, there was greater etoposide-induced linearization with yTOP2 full-length than with the two full-length human topoisomerases (Figure 6F). With the core enzymes, however, the effects of etoposide inverted, showing greatest stimulation of linearization for the hTOP2β core, then the hTOP2α core, followed by the yTOP2 core (Figure 9D). Curiously, etoposide also greatly increased the propensity of the hTOP2α core to nick DNA but had little such effect on the hTOP2β and yTOP2 cores (Figure 9E). By comparison, whereas vosaroxin induced cleavage by all three full-length enzymes (Figure 6A–D), only the hTOP2β core generated linear DNA products in the presence of vosaroxin, and even then at very low levels (Figure 9A–C) (as previously observed (36), vosaroxin-induced cleavage peaked at lower drug concentrations). Finally, as for the full-length enzymes (Figure 6A and B), no significant ciprofloxacin-induced linearization was observed with either the hTOP2α or hTOP2β core enzymes (Figure 9A and B), and the ciprofloxacin-induced linearization caused by yTOP2 full-length enzyme (Figure 6C) was reduced with yTOP2 core enzyme (Figure 9C). Collectively, these data show that the core catalytic region of human and yeast type II topoisomerases can be poisoned but that the presence of the ATPase domains in the full-length enzymes modulates drug-susceptibility of these core regions.

## DISCUSSION

Type IIA topoisomerases catalyze essential DNA unlinking events that can be corrupted by cytotoxic agents of clinical value. We have applied a large-scale mutagenesis and NGS analysis approach to better understand how eukaryotic type IIA topoisomerases discriminate between different classes of type IIA topoisomerase poisons. As a result, we accessed a global overview of residues frequently involved in drug discrimination that could be validated biochemically. In doing so, we also clarified the activity of vosaroxin and discovered that the susceptibility of the eukaryotic type IIA topoisomerase cleavage/re-ligation core domain to poisons is modulated by its ATPase domains.

### Type II topoisomerases can escape poisoning through many routes

Our initial complementation studies built upon previous work with the temperature-sensitive yeast strain JN394_t2–4_ (41–43,45,60–64). All three topoisomerases tested here—budding yeast TOP2 and human TOP2α and TOP2β—were sensitive to vosaroxin and etoposide (Figure 2A). Interestingly, yTOP2 also proved to be sensitive to ciprofloxacin (Figure 2A). This finding is in accord with prior biochemical studies that to our minds have been overlooked in the field (57,58), and suggest that it may be possible to repurpose antibacterial fluoroquinolones as inhibitors of fungal diseases. In this regard, ciprofloxacin acts synergistically with non-fluoroquinolone antifungals (65,66), but more potent fluoroquinolones, such as moxifloxacin, have yet to be assessed.

To attempt to more broadly define the potential resistance landscape to type II topoisomerase poisons, we conducted a large screen for drug-resistant topoisomerase II alleles using an NGS approach. The results of the NGS screen were corroborated by multiple factors including: (i) their overlap with hits obtained from our small-scale traditional genetic screen (Figures 2 and 3) and a number of mutations identified in previously published work (Supplementary Table S1) (42,45,63,64), and (ii) the validation of NGS-identified mutant phenotypes *in vivo* (Figure 5) and *in vitro* (Figures 7 and 8). This NGS screening methodology dramatically increases both the final total number of mutants that can be found in a screen and the speed at which they can be identified. We envision that NGS screens could be further improved using a higher number of transformants and additional mutagenesis strategies beyond hydroxylamine treatment (which favors GC→AT transitions in DNA (67)). As NGS platforms continue to develop, long-read sequencing, such as Single Molecule Real-Time Sequencing (Pacific Biosciences) or nanopore sequencing (Oxford Nanopore Technologies), could also be used to capture drug-resistant variants arising from more than one mutation in the topoisomerase gene.

Our NGS screens identified many mutations at widely-differing loci that conferred similar drug resistance phenotypes. We also observed multiple changes at a single locus, and changes within and outside the known quinolone- and epipodophyllotoxin-binding sites, that can give rise to selective resistance profiles (e.g. the A484T, A484V and A484G alterations in yTOP2) (Figure 3). Quinolone-resistance mutations have previously been identified far from drug-binding sites (68) (Supplementary Table S1). The emergence of resistance mutations throughout the entirety of eukaryotic topoisomerase II (Figure 4A) demonstrates that the prospective means for avoiding inhibition by type II topoisomerase poisons are broad and complex. This behavior differs from the typical drug-resistance outcomes that more specifically target residues near the site of drug action, such as with β-lactamases (69). Although mutations mapping to the nucleolytic core are found throughout the region, a degree of clustering was evident around the drug-binding regions for quinolones (27,28,70) and etoposide (25) (Figure 4C and Supplementary Figure S6B). A second cluster of mutations was also seen in a flexible loop region (residues A830-I841 in yTOP2) bearing a conserved isoleucine, I833, which intercalates into and bends DNA (71). Although changes to this isoleucine were not found within the top 100 hits of any of our screens (and would not be expected to, as the integrity of this position is needed for type IIA topoisomerase activity (72)), mutations that map to this loop include yTOP2^G832S^, the top Cip^R^Vos^R^ hit, which is adjacent to the intercalative isoleucine. A surprisingly high number of mutations were also found within the N-terminal ATPase domains, such as yTOP2^V197M^ (Cip^R^Vos^S^) (Figure 3A), which would be expected to alter ATP turnover by modifying the ATP-binding pocket. This observation suggests that a mechanistic link exists between ATP turnover and drug sensitivity, although the impact of this linkage differs significantly between topoisomerase homologs (as seen by the lower levels of mutations found in the ATPase domain of hTOP2α in comparison to yTOP2, Figure 4A). Altogether, the observed patterns of mutations in the nucleolytic core and ATPase domains suggest that binding and bending of the cleaved-DNA segment, which is coupled to the stimulation of ATP hydrolysis and strand passage (72), can have a profound effect on the potency and selectivity of type II topoisomerase poisons. Lastly, multiple drug-resistance mutations were identified in the unstructured C-terminal domains (Figure 4A). How these mutations would confer resistance is not immediately clear, but could impact catalytic activity, nuclear localization or both. Drug selectivity between topoisomerases therefore appears to be dictated by factors beyond just the favorability of the compound binding to the active site. Selectivity is instead likely to also include internal aspects such as conformational dynamics, overall catalytic rate and target DNA sequence and structure, along with extrinsic factors such as binding partners, cellular localization and local concentration. Focused studies involving different types of assays and different panels of mutants than those assessed here will be needed to more finely tease apart these couplings.

In some instances, our *in vitro* tested mutants had altered supercoil relaxation activity (Supplementary Figure S8). In particular, a reduction of topoisomerase activity can confer drug resistance (73), as was the case for hTOP2α^L926F^ (Supplementary Figure S8A). By contrast, both hTOP2α^P716H^ and yTOP2^A484V^ did not have altered relaxation activity compared to WT, whereas yTOP2^G832S^ had increased activity (Supplementary Figure S8). This result neatly demonstrates that there are multiple routes by which drug resistance can arise which may or may not directly impact catalytic function.

### Quinolone activity against eukaryotic type IIA topoisomerases can be modulated

Fluoroquinolones constitute one of the most popular treatments for bacterial infections and form the basis of a multi-billion dollar-per-year industry (31). Part of the success of fluoroquinolone-class drugs derives from their ability to preferentially act against prokaryotic type IIA topoisomerases such as gyrase and topoisomerase IV across a broad spectrum of bacteria (74), and because they are generally well-tolerated (75). Understanding the mechanism of fluoroquinolone specificity is important, not only as there is much debate over their side-effects (76), but also because quinolone variants that act against eukaryotic topoisomerase IIs are prospective anti-fungal and anti-cancer agents. Vosaroxin is one such quinolone variant known to have fewer toxic side-effects than other poisons, such as reduced reactive oxygen species production (36). Initial studies on vosaroxin demonstrated its activity against eukaryotic topoisomerase IIs (36,77), suggesting a potential use in treating AML (78). Thus far, vosaroxin has passed through phase 2 clinical trials (79) and has shown cautiously positive Phase 3 trial results in patients with relapsed or refractory AML (37). Vosaroxin is thought to have two activities against topoisomerase II: poisoning, which leads to DNA breaks, and the general inhibition of DNA supercoil relaxation (36). An apparent ability of vosaroxin to block negative supercoil relaxation was seen for all three enzymes tested (Figure 6); however, upon further investigation, we discovered that the retention of supercoiled substrate observed at increasing vosaroxin concentrations actually derived from topoisomerase II acting upon DNA supercoils formed by the intercalation of the drug into DNA (Supplementary Figure S7). Mutations that gave rise to vosaroxin resistance reduced the levels of DNA cleavage induced by the drug, but had no effect on this supercoiling activity (Figures 7 and 8). These data indicate that the cytotoxic behavior of vosaroxin arises primarily from its ability to poison topoisomerase II and stimulate dsDNA break formation, and not from a general inhibition of enzyme activity, as previously highlighted for other poisons (41,80). This separation should be considered when studying purported inhibitors of type II topoisomerases that are also DNA intercalating agents.

As might have been expected, the nucleolytic cores of the tested topoisomerase II enzymes remained susceptible to etoposide poisoning (Figure 9). This response manifested as dose-dependent linearization by all three core enzymes, but also as significant nicking by the hTOP2α core (Figure 9E). In addition, the sensitivity profiles of the core enzymes frequently differed from that of their full-length counterparts. For instance, yTOP2 displayed reduced ciprofloxacin-susceptibility when liberated from its ATPase elements and unstructured C-terminal tail (Figure 9C) compared to the full-length enzyme (Figure 6C). Second, although all full-length topoisomerase IIs proved susceptible to vosaroxin poisoning (Figure 6A–D), only the nucleolytic core of hTOP2β proved sensitive to vosaroxin on its own (Figure 9B). These observations not only corroborate the NGS data showing that the ability of poisons to act against type II topoisomerases can be modulated by regions outside of the principle drug binding locus, they also demonstrate that ATPase status, which controls DNA cleavage behavior (29,81), can be linked to drug efficacy and specificity. Interestingly, our data additionally highlight that the cores of hTOP2α and hTOP2β respond significantly differently to poisoning. Gaining a better understanding of the principles defining these behavioral differences will aid in developing isoform-specific inhibitors as cancer therapeutics with reduced off-target toxicity.

## DATA AVAILABILITY

Data files available on request.

## Supplementary Material

gkz579_Supplemental_Files

## Notes

Present address: Tim R. Blower, Department of Biosciences, Durham University, Durham, DH1 3LE, UK.

Present address: Amy S. Y. Lee, Department of Biology, Brandeis University, Waltham, MA 02453, USA.
