# Supplementary material for: A complex suite of loci and elements in eukaryotic type II topoisomerases determine selective sensitivity to distinct poisoning agents
Source: Nucleic Acids Res. 2019 Jul 9;47(15):8163–79. doi: 10.1093/nar/gkz579 (PMC6735899; doi:10.1093/nar/gkz579)
Supplement: gkz579_Supplemental_Files [file gkz579_supplemental_files.zip › Blower Bandak et al SuppFigs_with_legends.pdf]

Fig. S1

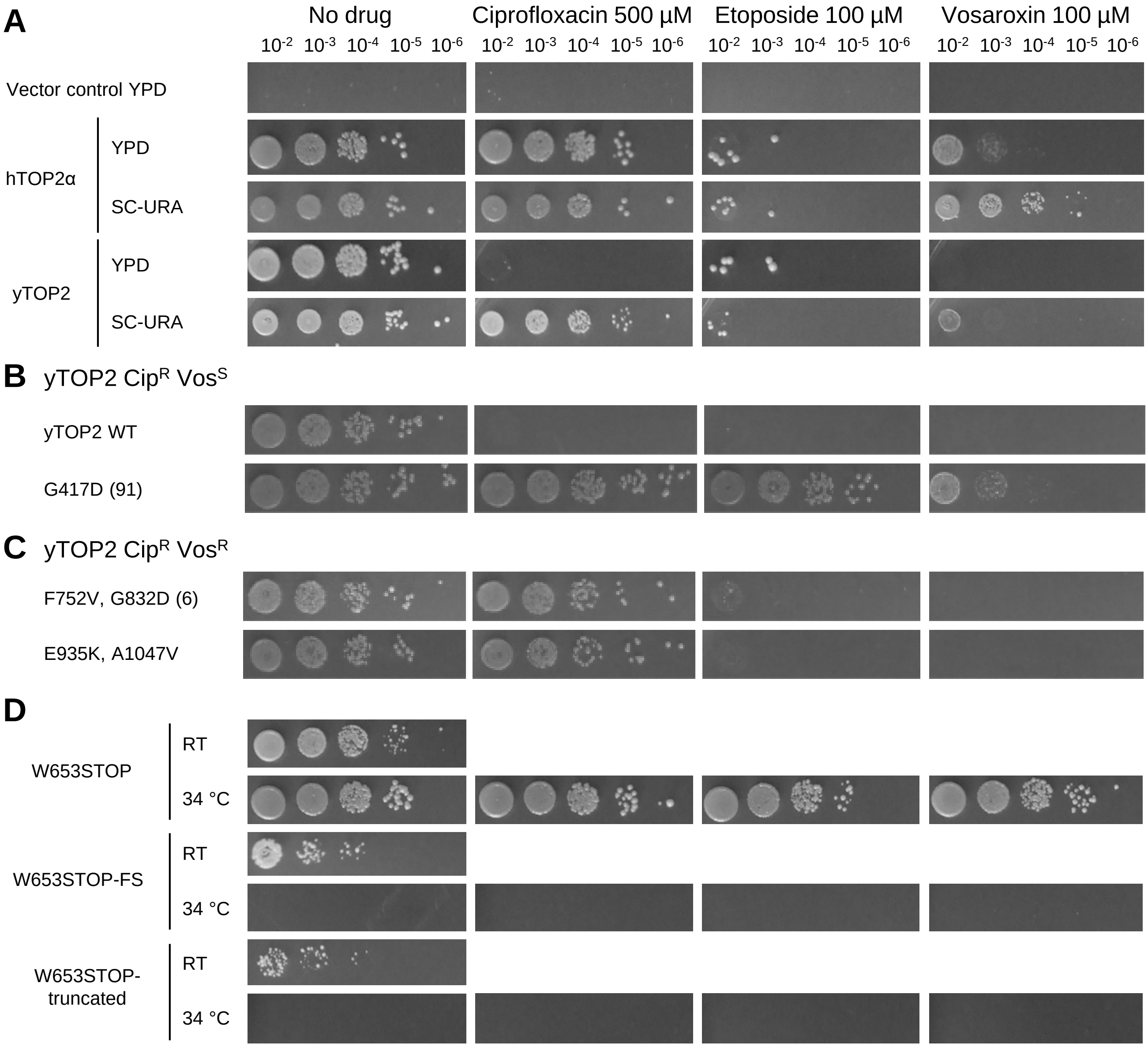

Fig. S1. Detail of growth conditions and selected mutants within the yeast screening procedure. (A) Growth media can widely alter the effect of topoisomerase poisons. Viable counts of JN394$_{t2\text{-}4}$ containing complementation constructs and plated at non-permissive temperature (34 °C), in the absence and presence of topoisomerase inhibitors. The dilution series of the strains carrying hTOP2α and yTOP2 have been plated on both YPD and SC-URA media. Although the effect of etoposide does not vary, both vosaroxin and ciprofloxacin are less effective on SC-URA. (B) and (C) Three mutants obtained in initial yTOP2 screens demonstrated an opposing phenotype to that expected from the selection and screening method applied. This prompted additional screening steps to be used in the larger NGS screen. (D) Transcriptional read-through provides drug resistance. Viable counts of JN394$_{t2\text{-}4}$ containing complementation constructs designed to investigate the W653STOP mutation were plated at both permissive (room temperature) and non-permissive temperature (34 °C), in the absence and presence of topoisomerase inhibitors. While the yTOP2 W653STOP mutation allows full complementation and resistance to all three topoisomerase poisons, constructs that contain W653STOP followed by a frame-shift (FS) or removal of the downstream ORF (truncated) grew poorly and could not complement.

Fig. S2

Perform hydroxylamine mutagenesis of plasmids pMJ1 (hTOP2α) and pTRB318 (yTOP2)

↓

Transform JN394$_{t2-4}$ with mutant plasmid pools

↙ ↘

Select Vos^{R} hTOP2α mutants by plating transformants on YPDA supplemented with 100 µM vosaroxin and incubating at 34 °C for three days

Select Cip^{R} yTOP2 mutants by plating transformants on YPDA supplemented with 500 µM ciprofloxacin and incubating at 34 °C for three days

↓

Transfer resulting colonies onto a YPDA plate supplemented with 500 µM ciprofloxacin and a second plate supplemented with 100 µM vosaroxin. Incubate at 34 °C for three days

↓

Transfer hTOP2α Vos^{R}, yTOP2 Cip^{R}Vos^{S} and yTOP2 Cip^{R}Vos^{R} colonies onto YPDA plates in groups of ~100. Incubate at 34 °C for three days

↓

For each individual plate, pool and re-suspend the colonies in sterile water

↓

Extract total DNA from each of the plate samples

↓

Use PCR to amplify the topoisomerase II ORFs from each of the total DNA samples

↓

Analyse the PCR reactions using agarose gel electrophoresis and extract the amplicons

↓

Prepare three libraries for Illumina sequencing by pooling the purified amplicons from the hTOP2α Vos^{R}, yTOP2 Cip^{R}Vos^{S} and yTOP2 Cip^{R}Vos^{R} samples

↓

Sequence the three libraries on a HiSeq2000 (Illumina)

↓

Perform quality analysis of sequence outputs. Map nucleotide mutations to amino acid changes. Rank mutations as top 100 by number of reads

↓

Perform site-directed mutageneses of plasmids pMJ1 and pTRB318 to re-make mutations of interest

↓

Transform JN394$_{t2-4}$ with mutant plasmids to confirm drug-resistance phenotype (Fig. 5)

↓

Express and purify mutant topoisomerase IIs for biochemical testing (Figs. 7 and 8)

Fig. S2. Flowchart of stages in the NGS screen.

Fig. S3

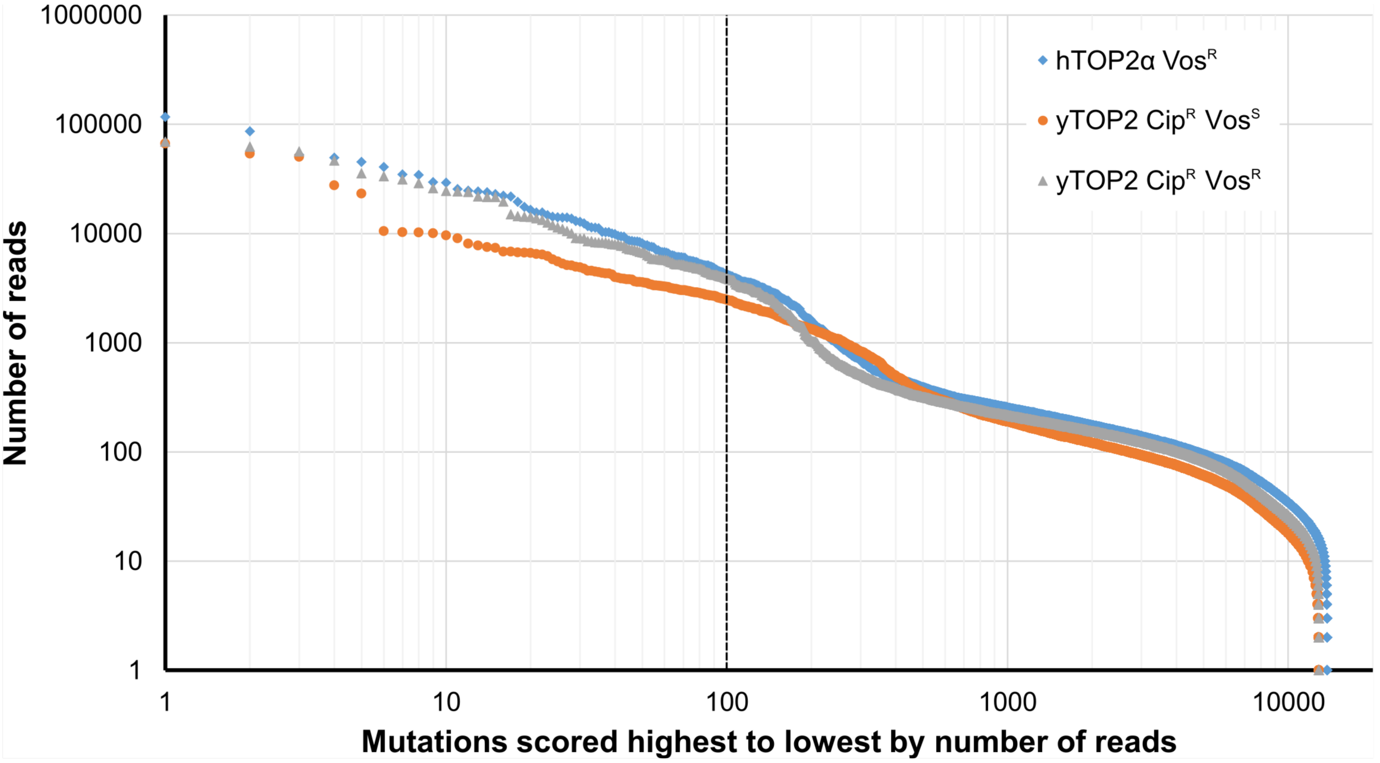

Fig. S3.Distribution of reads from all hits found within the three NGS screens. The conservative cut-off at 100 hits is indicated by a dashed line.

Fig. S4

x=Position hit in hTOP2α vosaroxin-resistant screen
x=Position hit in yTOP2 ciprofloxacin-resistant, vosaroxin-sensitive screen
x=Position hit in yTOP2 ciprofloxacin-resistant, vosaroxin-resistant screen
x=Position hit in both yTOP2 screens (though amino acid substitution may vary)
|=Starting residue of indicated protein domain, as determined using PDB entry 4FM9

```
                ATPase
yTOP2           |--------------------------------------MSTEPVSASDKYQKISQLEHIL       22
hTOP2α          |--------------MEVSPLQPVNE--NMQVNKIKKNEDAKKRLSVERIYQKKTQLEHIL       44
hTOP2β          |MAKSGGCGAGAGVGGGNGALTWVNNAAKKEESETANKNDSSKKLSVERVYQKKTQLEHIL       60
                |                                       :.: :*..  *** :******

yTOP2           KRPDTYIGSVETQEQLQWIYDEETDCMIEKNVTIVPGLFKIFDEILVNAADNKVRDPSMK        82
hTOP2α          LRPDTYIGSVELVTQQMWVYDEDVG-INYREVTFVPGLYKIFDEILVNAADNKQRDPKMS        103
hTOP2β          LRPDTYIGSVEPLTQFMWVYDEDVG-MNCREVTFVPGLYKIFDEILVNAADNKQRDKNMT        119
                 **********   *  *:***:.. :  ::**:****:************** **  .*.

yTOP2           RIDVNIHAEEHTIEVKNDGKGIPIEIHNKENIYIPEMIFGHLLTSSNYDDDEKKVTGGRN        142
hTOP2α          CIRVTIDPENNLISIWNNGKGIPVVEHKVEKMYVPALIFGQLLTSSNYDDDEKKVTGGRN        163
hTOP2β          CIKVSIDPESNIISIWNNGKGIPVVEHKVEKVYVPALIFGQLLTSSNYDDDEKKVTGGRN        179
                 * *.*. *.. *.: *:*****:  *: *::*:* :***:*******************

yTOP2           GYGAKLCNIFSTEFILETADLNVGQKYVQKWENNMSICHPPKITSYKKGPSYTKVTFKPD        202
hTOP2α          GYGAKLCNIFSTKFTVETASREYKKMFKQTWMDNMGRAGEMELKPF-NGEDYTCITFQPD        222
hTOP2β          GYGAKLCNIFSTKFTVETACKEYKHSFKQTWMNNMMKTSEAKIKHF-DGEDYTCITFQPD        238
                ************:* :***  :  : : *.* :**      ::. : .* .** :**:**

yTOP2           LTRFGMKELDNDILGVMRRRVYDINGSVRDINVYLNGKSLKIRNFKNYVELYLKSLEKKR        262
hTOP2α          LSKFKMQSLDKDIVALMVRRAYDIAGSTKDVKVFLNGNKLPVKGFRSYVDMYLKDKLDET        282
hTOP2β          LSKFKMEKLDKDIVALMTRRAYDLAGSCRGVKVMFNGKKLPVNGFRSYVDLYVKDKLDET        298
                *::* *:.**:**:.:* **.**: ** : ::* :**:.* :. *:.**::*:*.  .:

yTOP2           QLDNGEDGAAKSDIPTILYERINNRWEVAFAVSDISFQQISFVNSIATTMGGTHVNYITD        322
hTOP2α          -----------GNSLKVIHEQVNHRWEVCLTMSEKGFQQISFVNSIATSKGGRHVDYVAD        331
hTOP2β          -----------GVALKVIHELANERWDVCLTLSEKGFQQISFVNSIATTKGGRHVDYVVD        347
                           .   .:::*  *.**:*.:::*: .************: ** **:*:.*

yTOP2           QIVKKISEILKKKKKK--SVKSFQIKNNMFIFINCLIENPAFTSQTKEQLTTRVKDFGSR        380
hTOP2α          QIVTKLVDVVKKKNKGGVAVKAHQVKNHMWIFVNALIENPTFDSQTKENMTLQPKSFGST        391
hTOP2β          QVVGKLIEVVKKKNKAGVSVKPFQVKNHIWVFINCLIENPTFDSQTKENMTLQPKSFGSK        407
                *:* *: :::***:*   :** .*:**.:::*:*.*****:* *****::* : *.***
                                                       TOPRIM
yTOP2           CEIPLEYINKIMKTDLATRMFEIADANEEN-ALKKSDGT|RKSRITNYPKLEDANKAGTKE       439
hTOP2α          CQLSEKFIKAAIGCGIVESILNWVKFKAQVQLNKKCSAV|KHNRIKGIPKLDDANDAGGRN       451
hTOP2β          CQLSEKFFKAASNCGIVESILNWVKFKAQTQLNKKCSSV|KYSKIKGIPKLDDANDAGGKH       467
                *::  ::::      :.  ::: .. : :    **....|: .:*.  ***:***.** :.

yTOP2           GYKCTLVLTEGDSALSLAVAGLAVVGRDYYGCYPLRGKMLNVREASADQILKNAEIQAIK        499
hTOP2α          STECTLILTEGDSAKTLAVSGLGVVGRDKYGVFPLRGKILNVREASHKQIMENAEINNII        511
hTOP2β          SLECTLILTEGDSAKSLAVSGLGVIGRDRYGVFPLRGKILNVREASHKQIMENAEINNII        527
                . :***:******* :***:**.*:*** ** :*****:******* .**::****: *

yTOP2           KIMGLQHRKKYEDTK---SLRYGHLMIMTDQDHDGSHIKGLIINFLESSFPGLLDIQGFL        556
hTOP2α          KIVGLQYKKNYEDEDSLKTLRYGKIMIMTDQDQDGSHIKGLLINFIHHNWPSLLR-HRFL        570
hTOP2β          KIVGLQYKKSYDDAESLKTLRYGKIMIMTDQDQDGSHIKGLLINFIHHNWPSLLK-HGFL        586
                **:***::*.*:* .   :****::*******:********:***:. .:*.**  : **
                    Greek Key                                      TOPRIM
yTOP2           LEFI|TPIIKVSITKPTKNTIAFYNMPDYEKWREEESHKFTWKQKYYKGLG|TSLAQEVREY      616
hTOP2α          EEFI|TPIVKVSKN---KQEMAFYSLPEFEEWKSSTPNHKKWKVKYYKGLG|TSTSKEAKEY      627
hTOP2β          EEFI|TPIVKASKN---KQELSFYSIPEFDEWKKHIENQKAWKIKYYKGLG|TSTAKEAKEY      643
                 ***|***:*.* .   *: ::**.:*::::*:.   .:  ** *******|** ::*.:**
                      DNA-binding
yTOP2           FSNLDR|HLKIFHSLQGNDKDYIDLAFSKKKADDRKEWLRQYEPGT------------VLD       664
hTOP2α          FADMKR|HRIQFKYSGPEDDAAISLAFSKKQIDDRKEWLTNFMEDRRQRKLLGLPEDYLYG       687
hTOP2β          FADMER|HRILFRYAGPEDDAAITLAFSKKKIDDRKEWLTNFMEDRRQRRLHGLPEQFLYG       703
                *:::.*|*   *:    :*.  * ******: ******* ::                :
```

Fig. S4 continued

```
                                            Winged-Helix Domain
yTOP2           PTLKEIPISDFINKELILFSLADNIRSIPNVLD|GFKPGQRKVLYGCFKKNLKSELKVAQL      724
hTOP2α          QTTTYLTYNDFINKELILFSNSDNERSIPSMVD|GLKPGQRKVLFTCFKRNDKREVKVAQL      747
hTOP2β          TATKHLTYNDFINKELILFSNSDNERSIPSLVD|GFKPGQRKVLFTCFKRNDKREVKVAQL      763
                 : . :  .*********** :** ****.::*|*:********: ***:* * *:*****

yTOP2           APYVSECTAYHHGEQSLAQTIIGLAQNFVGSNNIYLLLPNGAFGTRATGGKDAAAARYIY       784
hTOP2α          AGSVAEMSSYHHGEMSLMMTIINLAQNFVGSNNLNLLQPIGQFGTRLHGGKDSASPRYIF       807
hTOP2β          AGSVAEMSAYHHGEQALMMTIVNLAQNFVGSNNINLLQPIGQFGTRLHGGKDAASPRYIF       823
                *  *:* ::***** :*  **: **********: ** * * ****  ****:*: ***:
                                                       DNA-binding
yTOP2           TELNKLTRKIFHPADDPLYKYIQEDEKTVEPEWYLPILP|MILVNGAEGIGTGWSTYIPPF      844
hTOP2α          TMLSSLARLLFPPKDDHTLKFLYDDNQRVEPEWYIPIIP|MVLINGAEGIGTGWSCKIPNF      867
hTOP2β          TMLSTLARLLFPAVDDNLLKFLYDDNQRVEPEWYIPIIP|MVLINGAEGIGTGWACKLPNY      883
                * *..*:* :*   **   *:: :*:: ******:**:*|*:*:**********:  :* :
                                                Tower
yTOP2           NPLEIIKNIRHLMNDEELEQMHPWFRGW|TGTIEEIEPLRYRMYGRIEQIGDNVLEITELP      904
hTOP2α          DVREIVNNIRRLMDGEEPLPMLPSYKNF|KGTIEELAPNQYVISGEVAILNSTTIEISELP      927
hTOP2β          DAREIVNNVRRMLDGLDPHPMLPNYKNF|KGTIQELGQNQYAVSGEIFVVDRNTVEITELP      943
                :  **::*:*:::  :   * * :: :|.***:*:   :* : *.:  :  ..:**:***

yTOP2           ARTWTSTIKEYLLL-GLSGNDKIKPWIKDMEEQHD-DNIKFIITLSPEEMAKTRKIGFYE       962
hTOP2α          VRTWTQTYKEQVLEPMLNGTEKTPPLITDYREYHTDTTVKFVVKMTEEKLAEAERVGLHK       987
hTOP2β          VRTWTQVYKEQVLEPMLNGTDKTPALISDYKEYHTDTTVKFVVKMTEEKLAQAEAAGLHK       1003
                .****.. ** :*   *.*.:*    *.* .* *   .:**::.:: *::*::.  *:::
                                Coiled-coil (towards Dimerization domain)
yTOP2           RFKLISPISLMNM|VAFDPHGKIKKYNSVNEILSEFYYVRLEYYQKRKDHMSERLQWEVEK      1022
hTOP2α          VFKLQTSLTCNSM|VLFDHVGCLKKYDTVLDILRDFFELRLKYYGLRKEWLLGMLGAESAK      1047
hTOP2β          VFKLQTTLTCNSM|VLFDHMGCLKKYETVQDILKEFFDLRLSYYGLRKEWLVGMLGAESTK      1063
                 *** : ::  .*|* **  * :***::* :** :*: :**.**  **: :   *  *  *
                                Dimerization domain
yTOP2           YSFQVKFIKMIIE|KELTVTNKPRNAIIQELENLGFPRFNKEGKPYYGSPNDEIAEQINDV      1082
hTOP2α          LNNQARFILEKID|GKIIIENKPKKELIKVLIQRGYDSDPVK---AWKEAQQK--------      1096
hTOP2β          LNNQARFILEKIQ|GKITIENRSKKDLIQMLVQRGYESDPVK---AWKEAQEK--------      1112
                 . *.:**   *:| :: : *: :: :*: * : *:     :    : . :::
                                                    Coiled-coil (away from Dimerization domain)
yTOP2           KGATSDEEDEESSHEDTENVINGPEELYGTYEYLLGMRIWSL|TKERYQKLLKQKQEKETE      1142
hTOP2α          --VPDEEEENEESDNEKETEKSDSVTDSGPTFNYLLDMPLWYL|TKEKKDELCRLRNEKEQE      1154
hTOP2β          --AAEEDET---QNQHDDSSSDSGTPSGPDFNYILNMSLWSL|TKEKVEELIKQRDAKGRE      1167
                  . .::*    ..:.  .  :.       ::*:* * :* *|***: ::* : :: *  *
                                                       CTD tail
yTOP2           LENLLKLSAKDIWNTDLKAFEVGYQEFLQRDAEARGGNV|PNKGSKTK-GKGKRKLVDDED      1201
hTOP2α          LDTLKRKSPSDLWKEDLATFIEELEAVEAKEKQDEQVGL|PGK-----GGKAKGKKTQMAE      1209
hTOP2β          VNDLKRKSPSDLWKEDLAAFVEELDKVESQEREDVLAGM|SGKAIKGKVGKPKVKKLQLEE      1227
                :: * : * .*:*: ** :*    : .  :: :     :|  *      ** * *  :  :

yTOP2           YDPSKKNKKSTARKGKKIKLEDKNFERILLEQKLVTKSKAPTKIKKEKTPSV-----SET       1256
hTOP2α          VLPSPRGQRVIPR-------------I----TIEMKAEAEKKNKKKIKN----------       1241
hTOP2β          TMPSPYGRRIIPE-------------I----TAMKA-DASKKLLKKKKGDLDTAAVKVE       1268
                  **   ::   .             *    .     .*  .*   *: .

yTOP2           KTEEEENAPSSTS-----SSSIFDIKKEDKDEGELSKISNKFKKISTIFDKMGSTSATSK       1311
hTOP2α          --ENTEGSPQEDGVELEGL-------KQRLEKKQKREPGTKTKKQTTLAFKP---IKKGK       1289
hTOP2β          FDEEFSGAPVEG-AGEEALTPSVPINKGPKPKREKKEPGTRVRKTPTSSGKPS--AKKVK       1325
                  *: . :* .               *    : :  : ..: :*  *   *      . *

yTOP2           ENTPEQDDVATKKNQTTAKKTAVKPKL---AKKPVRKQQKVVELSGESDLEILDSYTDRE       1368
hTOP2α          KRNPWSDSESDRSSDESN--FDVPPRETEP-RRAATKTKFTMDLDSDEDFSDFDEKT---       1343
hTOP2β          KRNPWSDDESKSESDLEETEPVVIPRDSLLRRAAAERPKYTFDFSEEDDDADDDDDDNN       1385
                :..* .*. :  ..:      * *:     :  . : : ..::. :.* .  *.

yTOP2           D-----------SNKDEDDAIPQRSRRQ-----RSSRAA---------SVPKKSYVETLE       1403
hTOP2α          ---------------DDEDFVPSDASP--------PKTKTSPKLSNKELKPQKSVVSDLE       1380
hTOP2β          DLEELKVKASPITNDGEDEFVPSDGLDKDEYTFSPGKSKATPEKSLHDKK----------       1435
                                ::: :*. .           ::
```

Fig. S4 continued

```
yTOP2           LS------------------DDSFIEDDEEENQGSDVSFNEED-----------------        1428
hTOP2α          ADDVKGSVPLSS-----SPPATHFPDE-------------TEITNPVPKKNVTVKKTAAK        1422
hTOP2β          SQDFGNLFSFPSYSQKSEDDSAKFDSNEEDSASVFSPSFGLKQTDKVPSKTVAAKKGKP-        1494
                 .                     * .:              :

yTOP2           ------------------------------------------------------------        1428
hTOP2α          SQSSTSTTGAKKRAAPKGTKRDPALN------SGVSQKPDPAKTKNR--RKRKPSTSD-D        1473
hTOP2β          ----SSDTVPKPKRAPKQKKVVEAVNSDSDSEFGIPKKTTTPKGKGRGAKKRKASGSENE        1550


yTOP2           ------------------------------------------------------------        1428
hTOP2α          SDSNFEKIVSKAV------TSKKSKGESDDFHMDFDSAVAPRAKSVRAKKPIKYLEESDE        1527
hTOP2β          GDYNPGRKTSKTTSKKPKKTSFDQDSDVDIFPSDFPTEPPSLPRTGRARKEVKYFAESDE        1610


yTOP2           -----------         1428
hTOP2α          DDLF-------         1531
hTOP2β          EEDDVDFAMFN         1621
```

Fig. S4. Sequence overview of the top 100 mutations for each screen. The protein sequences of yTOP2, hTOP2α and hTOP2β were aligned using Clustal Omega (48). Residues identified within the top 100 hits of each screen are highlighted. In the case of the yTOP2 screens, residues that were hit in both screens, though potentially with differing substitutions, are separately marked. The borders of protein domains from these type IIA topoisomerases are demarcated, as guided by PDB entry 4FM9. These borders have been used to generate the linear protein maps shown in Fig. 4.

Fig. S5

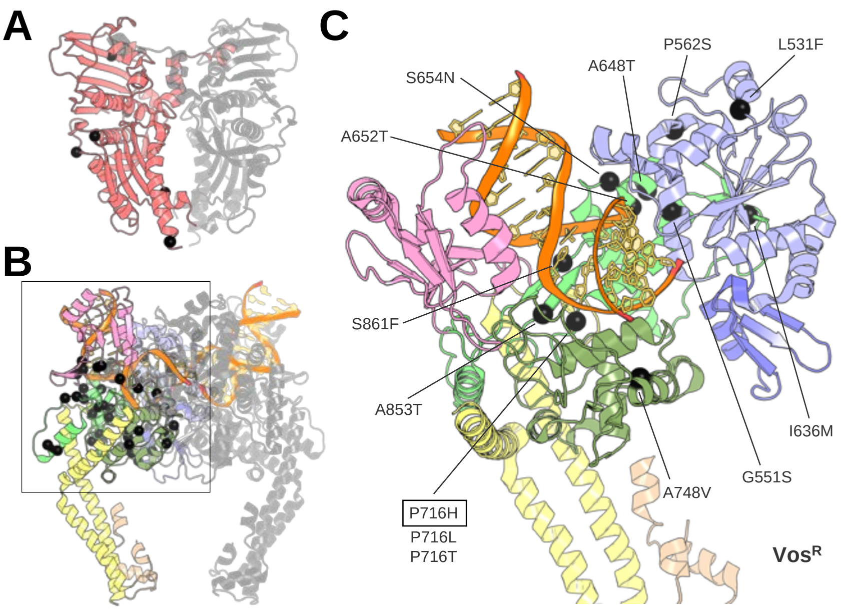

Fig. S5, related to Fig. 4. Vosaroxin resistance mutations in hTOP2α. Overview of the ATPase domain (A) and core enzyme (B) from hTOP2α, with one monomer colored as per Fig. 4A and the second monomer shown in transparent grey. DNA backbone and bases are shown in orange and wheat, respectively. Residues within these domains that were within the top 100 hits in the hTOP2α Vos^{R} screen are shown as black alpha-carbon spheres. (C) Close-up view of the boxed region in (B), with the partner protomer/DNA fragments removed for ease of visualization. Mutations identified within the top 100 hits of the hTOP2α Vos^{R} screen and tested in Figs. 2 and 5 are shown as per (A) and (B). The boxed mutation P716H was used for later *in vitro* testing. (A) was adapted from PDB entry 1ZXM; (B) and (C) were adapted from PDB entry 4FM9.

Fig. S6

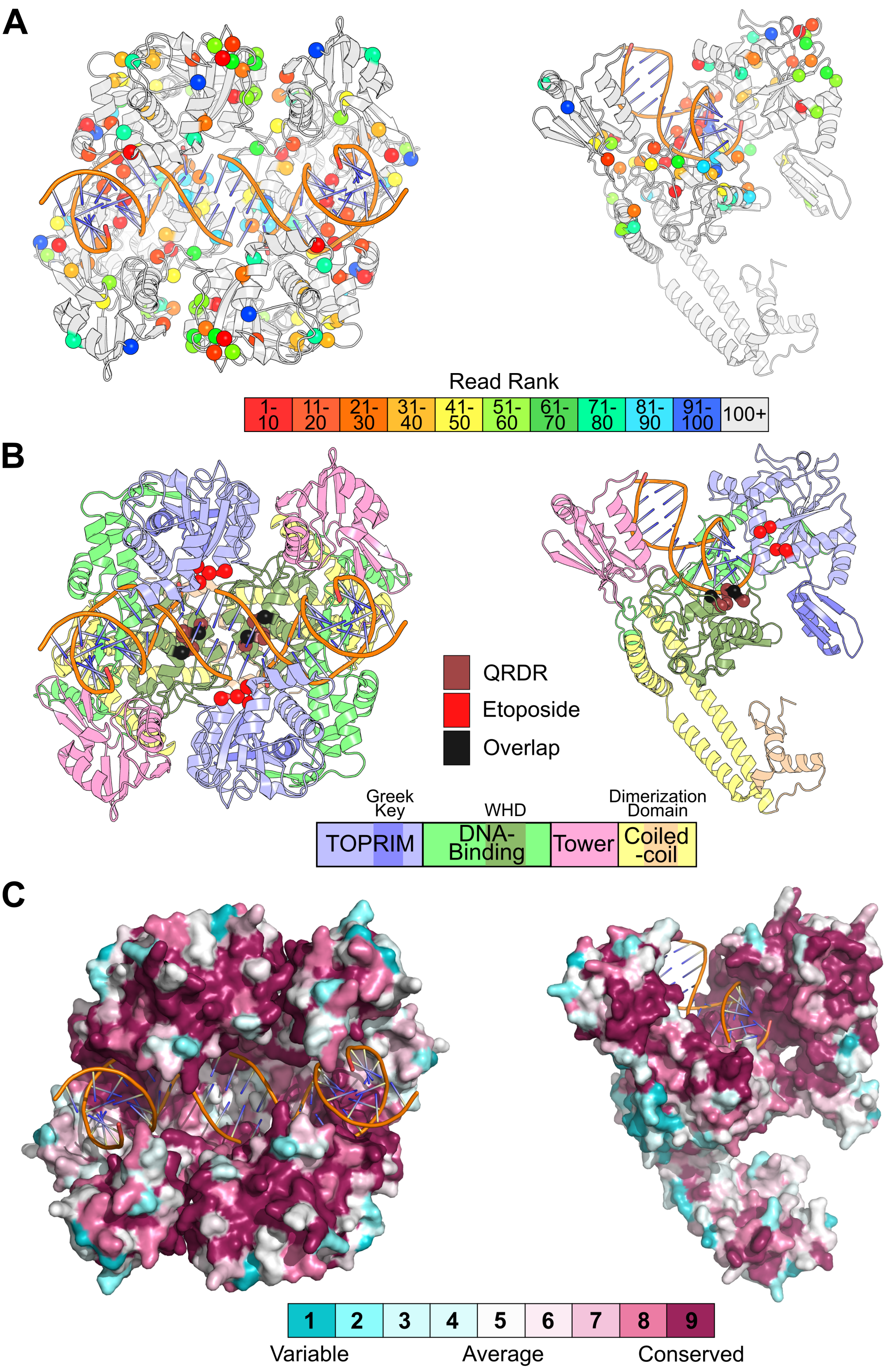

Fig. S6, related to Fig. 4. Functional analysis of drug-resistant mutation positions within the yTOP2 nucleolytic core enzyme. (A) Reads returned for each amino acid position of yTOP2 from both mutagenesis screens were summed and ranked. The top-scoring 100 amino acid positions identified by number of reads are coloredas alpha-carbon spheres in batches of ten, as indicated. Left-hand; a top-view of the core enzyme. Right-hand, a side view of a core monomer showing the protein-protein interface. (B) A top and side view as per (A), colored as per Fig. 4A. Residues within the region corresponding to the quinolone-resistance determining region of *E. coli* DNA gyrase (70) are shown as purple alpha-carbon spheres. Residues binding etoposide in hTOP2β (25) are shown as red alpha-carbon spheres. Residues implicated in both ciprofloxacin and etoposide binding are shown as an overlap in gray alpha-carbon spheres. (C) A top and side view of amino acid conservation within the yTOP2 core enzyme, as determined by Consurf (49), following multiple sequence alignment of homologous sequences from 51 independent species using Clustal Omega (48).

Fig. S7

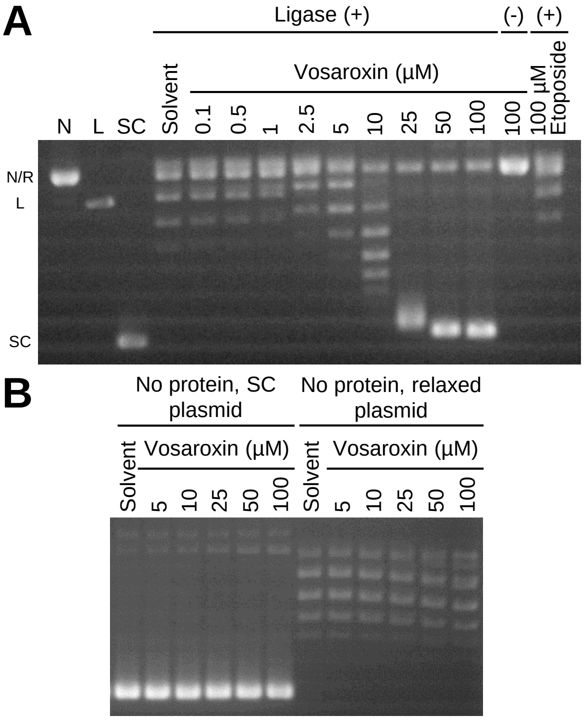

Fig. S7. Vosaroxin causes supercoiling of DNA. (A) A titration series of vosaroxin was incubated with nicked plasmid DNA and ligated with T4 DNA ligase. A no ligase control at 100 µM vosaroxin and a control with etoposide (which does not intercalate) and ligase were also performed. (B) Activity of vosaroxin on negatively supercoiled and relaxed plasmid DNA substrates in the absence of type II topoisomerases. Linear (L), nicked (N), relaxed (R) and supercoiled (SC) plasmids DNAs are provided as control lanes. The positions of linear (L), nicked (N), relaxed (R) and supercoiled (SC) plasmids DNAs are indicated.

Fig. S8

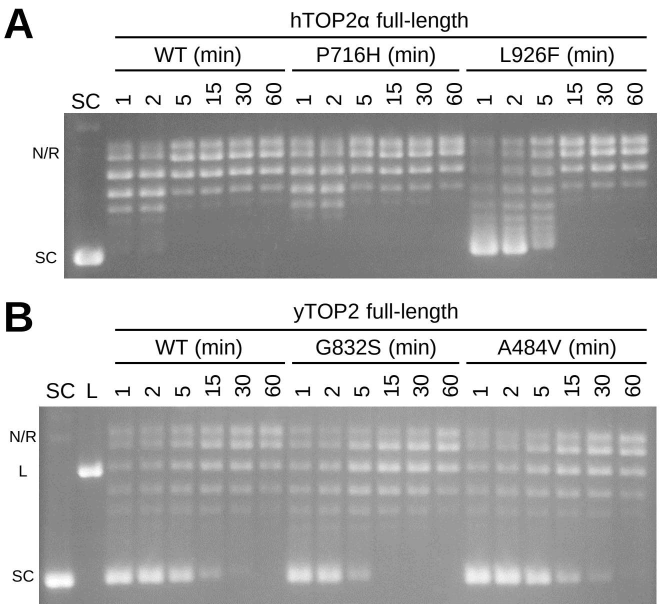

Fig. S8. Drug-resistance mutations can affect supercoil relaxation activity. (A) Time-courses of hTOP2α WT and two drug-resistant mutants hTOP2α^{P716H} and hTOP2α^{L926F} were performed using supercoiled plasmid DNA substrates. (B) Time-courses of yTOP2 WT and two drug-resistant mutants yTOP2^{G832S} and yTOP2^{A484V} were performed using supercoiled plasmid DNA substrates. Linear (L) and supercoiled (SC) plasmids DNAs are provided as control lanes. The positions of linear (L), nicked (N), relaxed (R) and supercoiled (SC) plasmids DNAs are indicated.
